# *In silico* single strand melting curve: a new approach to identify nucleic acid polymorphisms in Totiviridae

**DOI:** 10.1186/1471-2105-15-243

**Published:** 2014-07-16

**Authors:** Raffael AC Oliveira, Ricardo VM Almeida, Márcia DA Dantas, Felipe N Castro, João Paulo MS Lima, Daniel CF Lanza

**Affiliations:** Laboratório de Biologia Molecular Aplicada - LAPLIC, Departamento de Bioquímica, Centro de Biociências, Universidade Federal do Rio Grande do Norte, Natal, RN CEP: 59072-970 Brazil; Laboratório de Glicobiologia Molecular, Departamento de Bioquímica, Universidade Federal do Rio Grande do Norte, Natal, Brazil; Programa de Pós-Graduação em Sistemática e Evolução, Universidade Federal do Rio Grande do Norte, Natal, RN Brazil; Programa de Pós-Graduação em Bioquímica, Universidade Federal do Rio Grande do Norte, Natal, RN Brazil; Institute of Tropical Medicine of Rio Grande do Norte (IMT-RN), Universidade Federal do Rio Grande do Norte, Natal, RN Brazil

**Keywords:** RNA secondary structure, Infectious Myonecrosis Virus, high resolution melting curve, Virus detection, IHHNV, WSSV, Trichomonas

## Abstract

**Background:**

The PCR technique and its variations have been increasingly used in the clinical laboratory and recent advances in this field generated new higher resolution techniques based on nucleic acid denaturation dynamics. The principle of these new molecular tools is based on the comparison of melting profiles, after denaturation of a DNA double strand. Until now, the secondary structure of single-stranded nucleic acids has not been exploited to develop identification systems based on PCR. To test the potential of single-strand RNA denaturation as a new alternative to detect specific nucleic acid variations, sequences from viruses of the Totiviridae family were compared using a new *in silico* melting curve approach. This family comprises double-stranded RNA virus, with a genome constituted by two ORFs, ORF1 and ORF2, which encodes the capsid/RNA binding proteins and an RNA-dependent RNA polymerase (RdRp), respectively.

**Results:**

A phylogenetic tree based on RdRp amino acid sequences was constructed, and eight monophyletic groups were defined. Alignments of RdRp RNA sequences from each group were screened to identify RNA regions with conserved secondary structure. One region in the second half of ORF2 was identified and individually modeled using the *RNAfold* tool. Afterwards, each DNA or RNA sequence was denatured *in silico* using the softwares *MELTSIM* and *RNAheat* that generate melting curves considering the denaturation of a double stranded DNA and single stranded RNA, respectively. The same groups identified in the RdRp phylogenetic tree were retrieved by a clustering analysis of the melting curves data obtained from *RNAheat*. Moreover, the same approach was used to successfully discriminate different variants of *Trichomonas vaginalis* virus, which was not possible by the visual comparison of the double stranded melting curves generated by *MELTSIM*.

**Conclusion:**

*In silico* analysis indicate that ssRNA melting curves are more informative than dsDNA melting curves. Furthermore, conserved RNA structures may be determined from analysis of individuals that are phylogenetically related, and these regions may be used to support the reconstitution of their phylogenetic groups. These findings are a robust basis for the development of *in vitro* systems to ssRNA melting curves detection.

**Electronic supplementary material:**

The online version of this article (doi:10.1186/1471-2105-15-243) contains supplementary material, which is available to authorized users.

## Background

Despite the emergence of new techniques to nucleic acids investigation such as next generation sequence and array chips, the Polymerase Chain Reaction (PCR) and its variations still prevail in clinical laboratories. The use of PCR has grown increasingly in different applications ranging from microorganisms detection to diagnosis of complex genetic diseases [1–3]. The simple implementation and the possibility of post-PCR analysis automation make PCR a great tool for high throughput analysis [3]. Since its introduction with LifeCycler®, the post PCR low resolution melting analysis using SYBR® Green I dye is the method used to confirm the reaction specificity or to detect primer dimer formation and other non-specific products [4]. Some years later, the High Resolution Melting Analysis (HRMA) became possible with the advent of new intercalating dyes that could be used in high concentrations without compromising the PCR efficiency [5]. The HRMA technique allows fast high throughput analysis of PCR products and reinvigorated the use of DNA melting for a wide range of applications, including SNP genotyping and DNA mapping [6–9], gene scanning [10, 11], heterozygosity screening [12], species identification [13, 14] and many others.

The secondary structure formed by a particular nucleic acid molecule influences their DNA melting profile. Many bioinformatic tools designed to predict melting curves of nucleic acids are available [15–17]. Softwares that predict melting curves can efficiently validate regions with different denaturation profiles and these regions can be exploited to differentiate similar sequences and to define targets to post-PCR tests [18]. Until now, studies that attempt to develop molecular tools based on melting curves are restricted to denaturation of double-stranded DNA (dsDNA) molecules. Reports of secondary structures formed by a single nucleic acid strand, particularly single strand RNA (ssRNA), are focused in the determination of viral or viroid genome structures [19–22], noncoding RNAs (ncRNAs) and small interfering RNAs (iRNAs) [23–26].

Using carefully calculated thermodynamic parameters, algorithms can be used to predict the secondary structure of a RNA strand [27–33]. One of the most cited online servers that provide tools to work with RNA structures is the *Vienna RNA Package* [29]. Among the tools provided, *RNAfold* calculates the minimum free energy and predicts an optimal secondary structure using McCaskill’s algorithm [30]. *Vienna RNA Package* also provides the unique tool to assess ssRNA melting curves, the *RNAheat* software, which reads RNA sequences and calculates their specific heat in a determined temperature range, from the partition function by numeric differentiation [31–33].

The identification of RNA secondary structures is particularly interesting when viral genomes are analyzed. Previous studies demonstrated that conserved stem loops, extensive long-range interactions and small palindromic stem–loops generate structures that are generally associated with viral packing capacity and regulate viral replication [19, 21, 34]. However, such processes and mechanisms are not fully understood in Totiviridae family. Viruses of this family infect protozoa, fungi, insects and shrimps and some of these organisms have medical, zootechnical and agricultural importance [35–38]. Totiviridae members have monopartite double strand RNA (dsRNA) genomes organized in two ORFs. ORF1 encodes a capsid protein (CP) and ORF2 encodes an RNA-dependent RNA polymerase (RdRp) that is highly conserved among the family species [39].

In the present study we propose that the information extracted from a melting curve of a single stranded RNA molecule allows more precise detection of nucleotide variations than the traditional HRMA. To demonstrate our hypothesis, two softwares, *RNAheat* and *MELTSIM,* were used to generate melting curves of nucleic acid sequences from Totiviridae viruses. Melting curves generated were used to reconstruct groups determined by a traditional phylogenetic analysis, based on RdRp sequence alignment. Subsequently, ssRNA and dsDNA melting curves were compared for its potential to discriminate *Trichomonas vaginalis* virus isolates. Our results indicate that the information obtained by ssRNA denaturation may be used as a support to the development of more accurate methods to detect differences in nucleic acid sequences.

## Results and discussion

### Phylogenetic analysis of Totiviridae family

**Figure 1:**
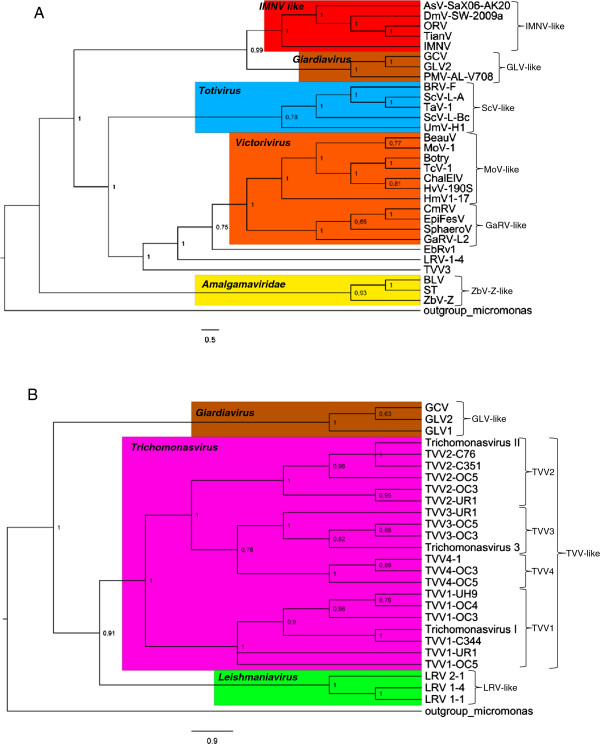
**Phylogenetic relationships between Totiviridae family members.** Trees were calculated from an alignment of RdRp aminoacid sequences from representative members of the Totiviridae family, using Bayesian inference. The IDs of the sequences in trees **A** and **B** are shown in Tables 1 and 2. The numbers in branch nodes indicate posterior probabilities. The right curly brackets indicate the groups identified in this study, named in accordance with Liu *et al*. [40] and de colors represent the genera in according to ICTV.

RNA-dependent RNA polymerases sequences are conserved within members of the families Totiviridae and Chrysoviridae [40]. Hence they were used to estimate the phylogenetic relationships among these viruses. Twenty eight RdRp aminoacid sequences referenced inTable 1 and two sequences referenced in Table 2 were aligned, and their phylogenetic relationships are shown in Figure 1A. Eight monophyletic groups can be defined in the obtained dendogram, and they were named following Liu *et al*. classification [40]. The groups IMNV-like, which comprises viruses that infect arthropods, GLV-like and ScV-like matched with previously described inferences [40]. Four new groups were retrieved: MoV-like that comprising viruses that infect plants and fungi, TVV-like and LRV-like that comprises virus that infect human protozoan parasites, and GaRV-like comprising fungus viruses. To ensure the efficiency of the analysis, relationships between TVV-like, LRV-like and GLV-like groups and their integrants were determined using the sequences referenced in Table 2 in a second phylogenetic analysis showed in Figure 1B. All observed groups are in agreement with the classification proposed by International Committee on Taxonomy of Viruses (ICTV) [41]. GLV-like comprises viruses of the genus *Giardiavirus* and ScV-like comprises viruses of the genus *Totivirus*. The genus *Victorivirus* includes two gropus, MoV-like and GaRV-like. The genera *Leishmaniavirus* and *Trichomonasvirus* include groups LRV-like and TVV-like respectively. IMNV-like group appears as less derived group near to GLV-like and does is not classified by ICTV. The *Zygosaccharomyces bailii* virus (ZbV-Z) and two other related viruses isolated from plants and fungus clustered together to form a ZbV-Z-like clade, on a basal branch of the phylogenetic tree (Figure 1A). Indeed, this group was formerly referred as a primitive, less derived group, distantly related to Totiviruses, and includes virions with distinct genomic organization from this family. A new family Amalgamaviridae has been proposed to accommodate these three viruses [40].

**Table 1:** **Totiviridae aminoacid sequences used in this study grouped according to phylogenetic analysis**

Virus name	Accession No.	Abbreviation
**MoV-like**		
*Beauveria bassiana RNA virus 1*	CCC42235	BeauV
*Tolypocladium cylindrosporum virus 1*	YP_004089630	TcV-1
*Botryotinia fuckeliana totivirus 1*	YP_001109580	BotryV
*Helminthosporium victoriae virus 190S*	NP_619670	HvV-190S
*Chalara elegans RNA virus 1*	YP_024728	ChalElV
*Helicobasidium mompa totivirus 1-17*	NP_898833	HmV1-17
*Magnaporthe oryzae virus 1*	YP_122352.1	MoV-1
**IMNV-like**		
*Infectious myonecrosis virus*	AHY18670.1	IMNV
*Tianjin totivirus*	AFE02920.1	TianV
*Omono river virus*	BAJ21511.1	ORV
*Drosophila melanogaster totivirus SW-2009a*	YP_003289293.1	DmV-SW-2009a
*Armigeres subalbatus virus SaX06-AK20*	YP_003934934.1	AsV-SaX06-AK20
**GLV-like**		
*Piscine myocarditis virus AL V-708*	YP_004581250.1	PMV-AL V-708
*Giardia canis virus from China* (2883–5981)*	DQ238861.1	GCV
*Giardia lamblia virus* (2880–5978)*	NC_003555.1	GLV2
**ZbV-Z like**		
*Blueberry latent virus isolate AR* (936–3332)*	HM029248.1	BLV
*Southern tomato virus isolate Mexico-1*(1039–3327)*	EF442780.1	STV
*Zygosaccharomyces bailii virus Z*	NP_624325.1	ZbV-Z
**ScV-like**		
*Ustilago maydis virus H1* (735–6002)*	NC_003823.1	UmV-H1
*Saccharomyces cerevisiae virus L-BC* (La)	NP_042581.1	ScV L-BC
*Saccharomyces cerevisiae virus L-A*	NP_620495.1	ScV L-A
*Black raspberry virus F*	YP_001497151.1	BRV-F
*Tuber aestivum* virus 1	ADQ54106.1	TaV-1
**GaRV-like**		
*Sphaeropsis sapinea RNA virus 2*	AAD11603.1	SphaeroV
*Coniothyrium minitans RNA virus*	YP_392467.1	CmRV
*Epichloe festucae virus 1*	CAK02788.1	EpiFesV
*Gremmeniella abietina RNA virus L2*	AAT48885.1	GaRV-L2
**Other sequence**		
*Eimeria brunetti RNA virus 1*	NP_108651	EbRV-1

*Accession numbers correspond to nucleotide sequences of complete genomes. Numbers in brackets correspond to first and last nucleotides of RdRp coding sequences.

**Table 2:** **Aminoacid sequences of** ***Trichomonasvirus*** **,** ***Leishmaniavirus*** **and** ***Giardiavirus*** **used in this study grouped according to phylogenetic analysis**

Virus name	Accession no.	Abbreviation
**TVV4**		
*Trichomonas vaginalis virus 4 strain TVV4-1*	AED99796.1	TVV4-1
*Trichomonas vaginalis virus 4 strain TVV4-OC3*	AED99794.1	TVV4-OC3
*Trichomonas vaginalis virus 4 strain TVV4-OC5*	AED99798.1	TVV4-OC5
**TVV3**		
*Trichomonas vaginalis virus 3 strain TVV3-UR1*	AED99800.1	TVV3-UR1
*Trichomonas vaginalis virus 3 strain TVV3-OC5*	AED99804.1	TVV3-OC5
*Trichomonas vaginalis virus 3 strain TVV3-OC3*	AED99802.1	TVV3-OC3
*Trichomonas vaginalis virus 3*	NP_659390.1	Trichomonasvirus_3
**TVV2**		
*Trichomonas vaginalis virus 2 strain TVV2-OC3*	AED99808.1	TVV2-OC3
*Trichomonas vaginalis virus 2 strain TVV2-UR1*	AED99806.1	TVV2-UR1
*Trichomonas vaginalis virus 2 strain TVV2-OC5*	AED99810.1	TVV2-OC5
*Trichomonas vaginalis virus II*	NP_624323.2	Trichomonasvirus_II
*Trichomonas vaginalis virus 2 isolate C76*	AET81014.1	TVV2-C76
*Trichomonas vaginalis virus 2 isolate C351*	AET81016.1	TVV2-C351
**TVV1**		
*Trichomonas vaginalis virus*	NP_620730.2	Trichomonasvirus_I
*Trichomonas vaginalis virus 1 isolate C344*	AET81012.1	TVV1-C344
*Trichomonas vaginalis virus 1 strain TVV1-UH9*	AED99814.1	TVV1-UH9
*Trichomonas vaginalis virus 1 strain TVV1-OC4*	AED99818.1	TVV1-OC4
*Trichomonas vaginalis virus 1 strain TVV1-OC3*	AED99816.1	TVV1-OC3
*Trichomonas vaginalis virus 1 strain TVV1-UR1*	AED99812.1	TVV1-UR1
*Trichomonas vaginalis virus 1 strain TVV1-OC5*	AED99820.1	TVV1-OC5
**LRV-like**		
*Leishmania RNA virus 2 - 1*	NP_043465.1	LRV 2-1
*Leishmania RNA virus 1 - 4*	NP_619653.1	LRV 1-4
*Leishmania RNA virus 1 - 1*	NP_041191.1	LRV 1-1
**GLV-like**		
*Giardia canis virus from China*	ABB36743.1	GCV
*Giardia lamblia virus*	AAM77694.1	GLV1
*Giardia lamblia virus*	NP_620070.1	GLV2

### Identification of conserved RNA secondary structures and melting curves generation

In HRMA, nucleotide variations between two PCR products are detected comparing their melting curves. Although this approach has been successfully used to identify sequence polymorphisms [6–8], and to discriminate bacterial strains and viruses variants [13, 14, 42], it can be rather inconclusive in some cases. High-resolution instruments and expensive dyes are required to detect punctual mutations or in situations where is necessary to detect multiple mutations in a same sequence [43, 44]. Considering that ssRNA melting curve is closed related to the secondary structure assumed by a ssRNA molecule, we decided to investigate if a melting curve of a ssRNA is more informative than a melting curve generated from a dsDNA. For this, RNA sequences from Totiviridae viruses coding for RdRp proteins were inspected in order to identify regions with conserved secondary structures. Conserved regions were selected to avoid major structural variation between the sequences. Initially, RNA sequences referenced in Tables 3 and 4 were screened but conserved RNA structures common to all sequences were not found. Interestingly, the alignment of the sequences from each group individually revealed regions with high probability (>90%) to form conserved RNA structures in groups IMNV-like, GaRV-like and GLV-like. Members of the MoV-like group showed conserved regions only when analyzed in subgroups, BotryV, TcV-1 and HvV-190S showed regions with conserved RNA structure in the second half of ORF2 when taken together. The same was observed to MoV-like members BeauV and MoV-1 when analyzed individually (data not shown). The groups ZbV-Z-like, ScV-like, TVV-like and LRV-like do not show RNA conserved regions with high *RNAz* score, nevertheless, one conserved region of each group could be selected manually from alignments (data not show). It is clear that the similarity between sequences increases the chance of finding regions with conserved RNA structure. In agreement with phylogenetic trees showed in Figure 1, individuals that share a secondary RNA structure belongs to groups with shorter branches.

**Table 3:** **Totiviridae nucleotide sequences used in this study grouped according to phylogenetic analysis**

Virus name	Acession code (GI)	Abbreviation
**MoV-like**		
*Beauveria bassiana RNA virus 1* (2672–5176)	345108726	BeauV
*Tolypocladium cylindrosporum virus 1* (2604–5126)	315573168	TcV-1
*Botryotinia fuckeliana totivirus 1* (2631–5147)	134141995	BotryV
*Helminthosporium victoriae virus 190S* (2605–5112)	124484600	HvV-190S
*Chalara elegans RNA virus 1* (2619–4067)	48696977	ChalElV
*Helicobasidium mompa totivirus 1–17* (2563–5100)	33867950	HmV1-17
*Magnaporthe oryzae virus 1* (2818–5316)	54193767	MoV-1
**IMNV-like**		
*Penaeid shrimp infectious myonecrosis virus* (5241–7490)	459680256	IMNV
*Tianjin totivirus* (5319–7535)	380715048	TianV
*Omono river virus* (5202–7535)	307933349	ORV
*Drosophila melanogaster totivirus SW - 2009a* (4841–6706)	268053723	DmV-SW-2009a
*Armigeres subalbatus virus SaX06-AK20* (5145–7430)	309259994	AsV-SaX06-AK20
**GLV-like**		
*Piscine myocarditis virus AL V-708* (3114–5294)	336042307	PMV-AL V-708
*Giardia canis virus from China* (2883–5981)	78217291	GCV
*Giardia lamblia virus* (2880–5978)	20143439	GLV2
**ZbV-Z Like**		
*Blueberry latent virus isolate AR* (936–3332)	308097100	BLV
*Southern tomato virus isolate Mexico-1*(1039–3327)	133776995	STV
*Zygosaccharomyces bailii virus Z* (1106–3037)	20889374	ZbV-Z
**ScV-like**		
*Ustilago maydis virus H1*	20564172	UmV-H1
*Saccharomyces cerevisiae virus L-BC (La)* (1970–4561)	9627980	ScV L-Bc
*Saccharomyces cerevisiae* virus L-A (2351–4546)	20428567	ScV L-A
*Black raspberry virus F* (2565–5009)	157939583	BRV-F
*Tuber aestivum virus 1* (2169–4556)	312233874	TaV-1
**GaRV-like**		
*Sphaeropsis sapinea RNA virus 2* (2658–5135)	3808226	SphaeroV
*Coniothyrium minitans RNA virus* (2386–4875)	78762702	CmRV
*Epichloe festucae virus 1* (2568–5051)	94536498	EpiFesV
*Gremmeniella abietina RNA virus L2* (2599–5076)	49036574	GaRV-L2
**Other sequence**		
*Eimeria brunetti RNA virus 1*(2667–5321)	NP_108651	EbRV-1

*Accession codes correspond to nucleotide sequences of complete genomes. Numbers in brackets correspond to first and last nucleotides of RdRp coding sequences.*

**Table 4:** **Nucleotide sequences of** ***Trichomonasvirus*** **,** ***Giardiavirus*** **and** ***Leishmaniavirus*** **used in this study grouped according to phylogenetic analysis**

Virus name	Acession code (GI)	Abbreviation
**TVV4**		
*Trichomonas vaginalis virus 4 strain TVV4 -1* (2534–4782)	332015871	TVV4-1
*Trichomonas vaginalis virus 4 strain TVV4-OC3* (2535–4783)	332015868	TVV4-OC3
*Trichomonas vaginalis virus 4 strain TVV4-OC5* (2534–4782)	332015874	TVV4-OC5
**TVV3**		
*Trichomonas vaginalis virus 3 strain TVV3-UR1* (2448–4693)	332015877	TVV3-UR1
*Trichomonas vaginalis virus 3 strain TVV3-OC5* (2445–4690)	332015883	TVV3-OC5
*Trichomonas vaginalis virus 3 strain TVV3-OC3* (2449–4694)	332015880	TVV3-OC3
*Trichomonas vaginalis virus 3* (2645–4690)	21450040	Trichomonasvirus_3
**TVV2**		
*Trichomonas vaginalis virus 2 strain TVV2-OC3* (2380–4607)	332015889	TVV2-OC3
*Trichomonas vaginalis virus 2 strain TVV2-UR1* (2379–4606)	332015886	TVV2-UR1
*Trichomonas vaginalis virus 2 strain TVV2-OC5* (2378–4605)	332015892	TVV2-OC5
*Trichomonas vaginalis virus II* (2302–4605)	20889358	Trichomonasvirus_II
*Trichomonas vaginalis virus 2 isolate C76* (2317–4620)	357529890	TVV2-C76
*Trichomonas vaginalis virus 2 isolate C351* (2314–4617)	357529893	TVV2-C351
**TVV1**		
*Trichomonas vaginalis* virus (2308–4578)	20564174	Trichomonasvirus_I
*Trichomonas vaginalis virus 1 isolate C344* (2316–4578)	357529887	TVV1-C344
*Trichomonas vaginalis virus 1 strain TVV1-UH9* (2353–4615)	332015898	TVV1-UH9
*Trichomonas vaginalis virus 1 strain TVV1-OC4* (2353–4615)	332015904	TVV1-OC4
*Trichomonas vaginalis virus 1 strain TVV1-OC3* (2355–4617)	332015901	TVV1-OC3
*Trichomonas vaginalis virus 1 strain TVV1-UR1* (2354–4616)	332015895	TVV1-UR1
*Trichomonas vaginalis virus 1 strain TVV1-OC5* (2351–4613)	332015907	TVV1-OC5
**LRV-like**		
*Leishmania RNA virus 2–1* (2858–5191)	9628596	LRV 2-1
*Leishmania RNA virus 1–4* (2605–5241)	20153346	LRV 1-4
*Leishmania RNA virus 1–1* (2612–5236)	9626920	LRV 1-1
**GLV-like**		
*Giardia canis virus from China* (2883–5981)	78217291	GCV
*Giardia lamblia virus* (2880–5978)	20143439	GLV2
*Giardia lamblia virus* (2880–5978)	21780360	GLV1

*Accession codes correspond to nucleotide sequences of complete genomes. Numbers in brackets correspond to first and last nucleotides of RdRp coding sequences.*

**Figure 2:**
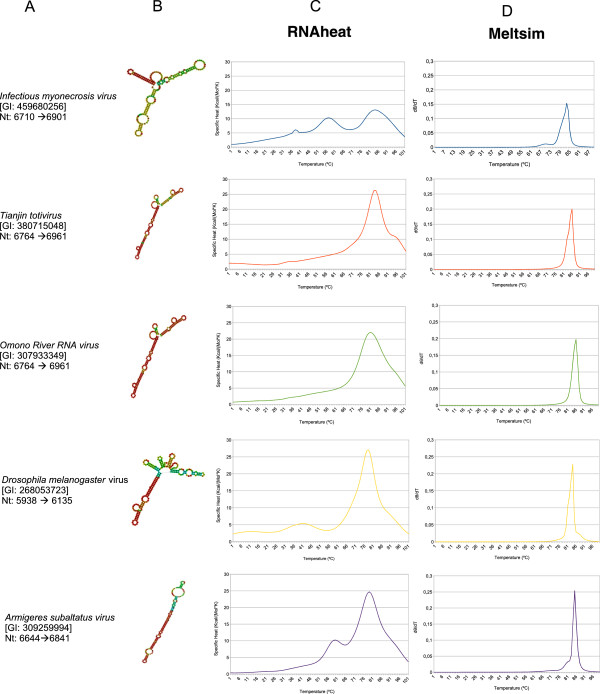
**Regions with conserved RNA secondary structures and their respective melting curves.** This figure corresponds to analysis of IMNV-like group sequences. **(A)** Indication of regions with conserved secondary structure inside RdRp coding sequences, identified using *RNAz*. **(B)** Minimum free energy models calculated using *RNAfold* corresponding to each conserved region identified by *RNAz.* Structures are colored according to base-pairing probabilities. Red color denotes the high probability and purple denotes low probability of a given base is paired or not. For unpaired regions the color denotes the probability of being unpaired. (**C** and **D**) Melting curves calculated from conserved regions using softwares *RNAheat* and *MELTSIM* respectively.

RNA secondary structures of the conserved regions found in groups IMNV-like, GLV-like and GaRV-like were predicted using the software *RNAfold.* RNA fragments that show conserved RNA secondary structures in IMNV-like group are indicated in Figure 2 column A. The respective models generated from each sequence are showed in Figure 2 column B. These sequences were also used to perform a *in silico* melting curve analysis using softwares *RNAheat* and *MELTSIM,* in order to obtain ssRNA melting curves (Figure 2 column C) and dsDNA melting curves (Figure 2 column D). The results of the same analysis from groups GLV-like and GaRV-like are showed in Additional file 1: Figure S1 and Additional file 2: Figure S2 respectively. Is interesting to observe that, in all cases, ssRNA melting curve presents higher variation than the profile generated by denaturation of dsDNA. This variation is possibly due to the presence of "bubbles" or “hairpins” formed as result of regions that not have perfect base pair complementarities. These regions may comprise several small pieces that present different melting temperatures. When dsDNA is used, the melting curve variation is generated only due to differences in the number of hydrogen bonds between the strands, which can be caused by nucleotide mispairing. This subtle variation in dsDNA melting curve can be detected only using more sensitive and expensive methods. Denaturation profile generated by ssRNA, as a result of the loss of its secondary structure, reflects more intense variations in nucleotide sequence unambiguously. These variations are more pronounced if the number of paired regions interspersed with non complementary regions is high. This can be easily observed when comparing the graphs of columns C and D in Figure 2. Is possible to distinguish five profiles in column C visually, but is not possible to do it comparing profiles that are in column D.

### Clustering groups using melting curves

**Figure 3:**
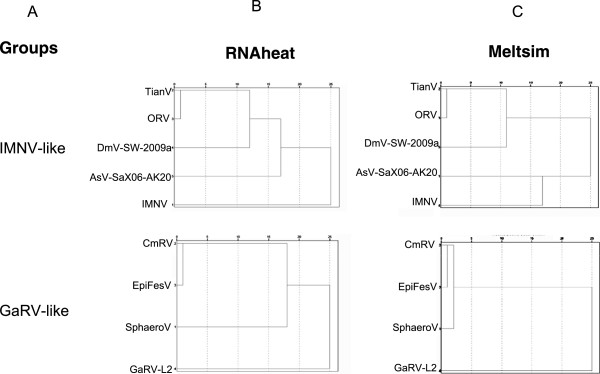
**Cluster analysis and dendograms of groups IMNV-like and GaRV-like.** Melting curves generated for each conserved RNA sequence in a same group were compared and clustered using a statistical inference. The proximity between individuals of groups indicated in the column **(A)** is due exclusively to the similarity between the melting curves generated *in silico*. Columns **(B)** and **(C)** shows the dendograms calculated from curves generated by *RNAheat* and *MELSTSIM* for the members of each group.

To confirm that information extracted from a ssRNA melting curve is more detailed than its correspondent dsDNA melting curve, clustering analyses were performed using melting curves from each ssRNA and dsDNA sequences of groups IMNV-like, GaRV-like and GLV-like. The curves were compared per group and clustered using R [45]. The results are represented as dendograms in Figure 3 and in Additional file 3: Figure S3. The relationships between individuals are determined exclusively by the similarity between the melting curves generated by the programs *RNAheat* and *MELTSIM*. The groups obtained from R analyses (Figure 3) were compared to groups obtained in phylogenetic analysis. It was surprising that the IMNV-like and GaRV-like groups could be perfectly reconstructed by the clustering of the *RNAheat* melting curves data, but not by the clustering based on *MELTSIM* melting curves. In these two cases, the analysis using ssRNA melting curves showed more resolution than the analysis using dsDNA melting curve. In other words, these results strongly indicate that ssRNA melting curve is a good source of information about the nucleic acid sequence. Additional tests using the conserved sequences manually selected from the other groups confirm that the resolution of dendrograms generated from *RNAheat* curves is never less than the resolution of dendograms generated from *MELTSIM* curves (data not show).

It is already known that the formation of secondary structures in DNA can exerts significant influence in the molecule functions during DNA replication, transcription or translation. These secondary structures may vary within the molecule or when DNA is transcribed to RNA in according to cellular context involved. Considering this fact, is perfectly plausible that a given nucleic acid sequence may suffer different selective pressures in according with variations of it conformation in different stages of its “life cyle”. In single stranded RNA viruses, the secondary structure of RNA could be selected by a large number of factors acting at the same time, including the compactation of the genetic material into capsid. Therefore, we opted to eliminate any noise that could compromise the analysis of RNA conserved secondary structures and ensure that natural selection would be acting mainly on the structure detected by *RNAz*. Due to this fact the Totiviridae family seems a perfect model. During all replication steps the genetic material of Totiviridae remains as RNA and the formation of RNA secondary structures occur only when RNA is being replicated. This factor can be decisive for the perfect reconstruction of phylogenetic groups comparing secondary structures of RNA.

### Potential of single strand melting curve to pathogen identification

**Figure 4:**
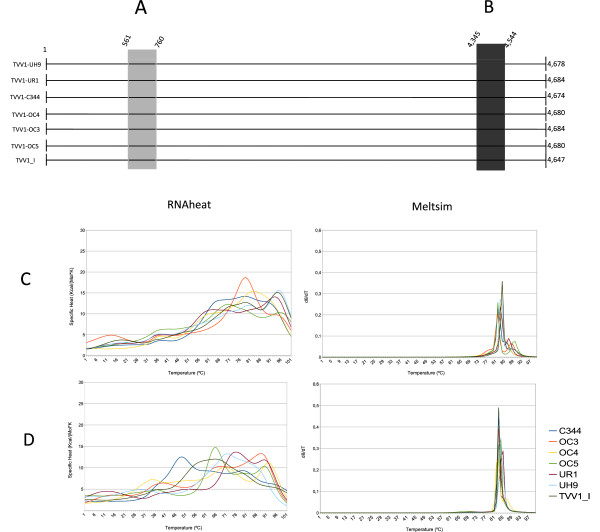
**Differentiating members of the subgroup TVV1 using** ***in silico*** **melting curves.** A region with variable RNA secondary structure **(A)** and a region with conserved RNA structure **(B)** were obtained from the alignment of ORF2 of all members of the group using the software *RNAz*. The curves were generated by *RNAheat* and *MELTSIM* to conserved regions **(C)** and variable regions **(D)**. Each denaturation curve is marked with a different color: Dark blue lines to TVV1-C344; orange lines to TVV1-OC3; yellow lines to TVV1-OC4; green lines to TVV1-OC5; dark red lines to TVV1-UR1; light blue lines to TVV1-UH9 and dark green lines to TVV1_I.

Whereas the analysis of single strand denaturation enables a higher resolution to phylogenetic groups reconstruction, it is expected to be also more efficient in distinguishing individuals within the same group. To confirm this, a phylogenetic analysis of sequences from a large number of members of TVV-like group was performed (Figure 1B). The analysis of different variants of *Trichomonas vaginalis* virus, revealed four five sub-groups called TVV2, TVV3, TVV4 and TVV1, all belonging to the group TVV-like and to genus *Trichomonasvirus*. The sub-group TVV1 was selected to generate melting curves *in silico*. An alignment of RdRp RNA sequences was used for *RNAz* screening. This analysis revealed one region with conserved RNA structure shared by all viruses of this group in the second half or RdRp RNA sequence. Then, two regions were selected, a non-conserved region and the conserved region detected by *RNAz* (Figure 4A and 4B, respectively). These regions were used to generate melting curves using *RNAheat* and *MELTSIM* (Figure 4C and 4D respectively). It was clear that the melting curves generated from ssRNA are more informative than the curves generated by denaturation of dsDNA. Observing the curves generated by *RNAheat* in both sets of melting curves is possible to differentiate seven *Trichomonasvirus* variants. The discrimination of each virus is more difficult if the graphs generated by *MELTSIM*, because the variation in the melting curves occurs in a restricted temperature range.

## Conclusions

The results presented here are a strong indication that the ssRNA melting curves are more informative than dsDNA melting curves. In addition, they demonstrate that common RNA conserved regions may be determined from analysis of individuals that are phylogenetically related, and that these regions may be used to support the reconstitution of their phylogenetic groups. These findings are a robust basis for the development of *in vitro* systems to ssRNA melting curves detection.

## Methods

### Data acquisition and phylogenetic analysis

The nucleotide and amino acid sequences from Totiviridae viruses were retrieved from public repositories such as GenBank [http://www.ncbi.nlm.nih.gov] and UniProt [http://www.uniprot.org]. Sequences were aligned using TCOFFEE and MCOFFEE algorithms [46] using default parameters, and manually edited using Jalview v. 2.8 [47]. ORFs and protein conserved domains identification were performed using ORF finder and NCBI conserved domain database (CDD), respectively. The RdRP sequence from *Micromonas pusilla virus* (Reoviridae family; Accession number YP654545) was used as outgroup due to its higher proximity and similarity to the family Totiviridae. The RdRP sequences were then aligned at the amino acid level, using the program MAFFT v. 6.85 [48, 49] with the L-INS-I parameter, gap opening penalty 1.53 and offset value 0.1, guided by the structural alignment from protein family pfam02123, present in the Conserved Domains Database (CDD) [50]. Then, they were re-aligned using the program Muscle [51]. Afterwards, the best-fit amino acid substitution model was estimated using ProtTest v.3.2 [52] and the dendograms were calculated based on a Bayesian analysis using MrBayes 3.1.2 [53, 54] and BEAST v.1.8 [55]. All indels and non-informative sites (missing data) in the alignment were treated as partial deletion, with a cutoff of 75%, to avoid potentially ambiguous regions in topologies. The Bayesian inferences were conducted using three independent runs, with fixed LG or WAG model, gamma distributed rates among sites and fixed amino acid frequencies. Each Markov Chain was initiated with a random tree and run for 10^6^ generations, sampled every 100 generations, and a consensus tree was estimated by using a burning in of 1,000,000 trees. The convergence of the simultaneous runs was assessed using the Tracer tool v. 1.5 [56], in order to evaluate the statistic support and robustness of the bayesian analysis. The trees generated by the programs were edited in the program FigTree [56].

### RNA secondary structure prediction

Conserved RNA secondary structures were detected from TCOFFEE multiple alignments of RdRp RNA sequences (Tables 3 and 4), using the *RNAz* software, provided by *Vienna RNA Web Services* [57]. This tool detects a consensus secondary structure for an alignment based on thermodynamic stability and structural conservation. A normalized measure of thermodynamic stability is computed by comparing the minimal free energy (MFE) of a native sequence to the MFEs of a large number of random sequences of the same length and base composition. Then, a *z-score* is calculated from the relation *z = (m -μ )/σ,* where *μ* and *σ* are the mean and standard deviations, respectively, of the MFEs of the random samples [58]. Negative *z-scores* indicate that a sequence is more stable than expected by chance. The structural conservation is predicted using the *RNAalifold* approach [59]. The secondary structures were then calculated using the sequences selected from the *RNAz* output using *RNAfold* software provided by *Vienna RNA Package* [57]. *RNAfold* reads RNA sequences, calculates their MFE structure and free energy. The *-p* option was used to compute the partition function (PF) and base pairing probability matrix, as well as the overall free energy of the thermodynamic ensemble. *RNAfold* produces PostScript files with plots of the resulting secondary structure graph and a dot plot of the base pairing matrix. Default parameters were used to generate the interactive RNA structure plots.

### Melting curve analysis

The dsDNA melting curves were estimated using the *MELTSIM* software, which generates derivative profiles. In the model used by this software, proposed by Blake *et al.* [15], the loop entropy has been appended in a one-dimensional Ising lattice [60–62]. By default, the program starts the simulation at 60°C (T1), increasing the temperature in every 0.050 degrees, until it reaches 100°C (T2). The single strand RNA melting curves were estimated using the *RNAheat* software [31]. This program reads RNA sequences and calculate their specific heat in a predetermined temperature range, from a partition function by numeric differentiation that describes the statistical properties of a system in thermodynamic equilibrium. The temperature dependence of the partition function gives information about the secondary structure melting behavior. The overwhelming majority of configurations are in the unfolded state and the high temperature ensemble is unfolded. According to reference point proposed by McCaskill [30] for the entropy of zero for an unfolded chain, the partition function must decrease toward one at high temperature and the specific heat reflects the occurrence of any structural transitions as the temperature increases. The result is written as a list of temperature degrees in °C versus specific heat in Kcal/(Mol * K) [31]. The results calculated from 0 to 100°C were plotted using R [45].

### Statistical and grouping analysis

Based on the melting denaturation scores, the melting curves were clustered using a hierarchical cluster analysis, using R [45]. This technique was used to identify the mutually exclusive groups that could be obtained based in the sample, considering only the similarities or differences between them. In this procedure, dendograms with the clusters were identified using the single linkage (nearest neighbor) method with the measure of Euclidean distance squared. This algorithm takes the two objects with the smallest distance and clusters them in the first group. Then, it takes the next object with the smallest distance and this third object is clustered with the first group, being included in the group a new group with two objects is obtained. This process keeps going until all objects are allocated to a group. The nucleotide sequences from the identified regions with conserved secondary structures were aligned in *MEGA5* [63] using the *MUSCLE* algorithm. Each alignment was used in a neighbor-joining grouping analysis, using Maximum-composite likelihood distance and 500 bootstrap replications. The obtained dendograms were visually compared to the ones from hierarchical cluster analyses, based on the single and double strand DNA melting denaturation cores.

### Availability of supporting data

All supporting data are included in Additional files.

## Electronic supplementary material

Additional file 1: Figure S1: Regions with conserved RNA secondary structures identified in GLV-like group and their respective melting curves. (A) Regions with secondary structures identified using *RNAz* software, from the alignment of ORF2 RNA sequences of GLV-like group members. (B) Secondary structure calculated using *RNAfold*, corresponding to each conserved region identified by *RNAz*. (C) Melting curves calculated from the conserved region, using the software *RNAheat* which considers ssRNA denaturation. (D) Melting curves calculated from the conserved region, using the software *MELTSIM* which considers dsDNA denaturation. (PPT 270 KB)

Additional file 2: Figure S2: Regions with conserved RNA secondary structures identified in GaRV-like group and their respective melting curves. (A) Regions with secondary structures identified using *RNAz* software, from the alignment of ORF2 RNA sequences of GaRV-like group members. (B) Secondary structure calculated using *RNAfold*, corresponding to each conserved region identified by *RNAz*. (C) Melting curves calculated from the conserved region, using the software *RNAheat* which considers ssRNA denaturation. (D) Melting curves calculated from the conserved region, using the software *MELTSIM* which considers dsDNA denaturation. (PPT 302 KB)

Additional file 3: Figure S3: Cluster analysis and dendogram of GLV-like group. The curves generated for each sequence were compared and clustered using a statistical inference. The proximity between individuals of groups indicated in the column (A) is due exclusively to the similarity between the melting curves generated *in silico*. Columns (B) and (C) shows the dendograms calculated from the curves generated by the programs *RNAheat* and *MELSTSIM* for the members of GLV group. (PPT 355 KB)

## Abbreviations

PCR: Polymerase chain reaction
ORF: Open reading frame
RdRP: RNA-dependent RNA polymerase
dsDNA: Double-stranded DNA
ssRNA: Single-stranded RNA
HRMA: High resolution melting analysis
SNP: Single nucleotide polymorphism
ncRNA: Non-coding RNA
siRNA: small interfering RNA
CP: Capsid protein
CDD: Conserved domain database
ML: Maximum likelihood
MP: Maximum parsimony
TBR: Tree bissection reconnection
NNI: Nearest neighbor interchange
MFE: Minimum free energy
PF: Partition function.
