# Supplementary material for: In silico single strand melting curve: a new approach to identify nucleic acid polymorphisms in Totiviridae
Source: BMC Bioinformatics. 2014 Jul 16;15(1):243. doi: 10.1186/1471-2105-15-243 (PMC4119202; doi:10.1186/1471-2105-15-243)
Supplement: Supplementary file 1 — Additional file 1: Figure S1: Regions with conserved RNA secondary structures identified in GLV-like group and their respective melting curves. (A) Regions with secondary structures identified using RNAz software, from the alignment of ORF2 RNA sequences of GLV-like group members. (B) Secondary structure calculated using RNAfold, corresponding to each conserved region identified by RNAz. (C) Melting curves calculated from the conserved region, using the software RNAheat which considers ssRNA denaturation. (D) Melting curves calculated from the conserved region, using the software MELTSIM which considers dsDNA denaturation. (PPT 270 KB) [file 12859_2013_6519_MOESM1_ESM.ppt]

## Slide 1
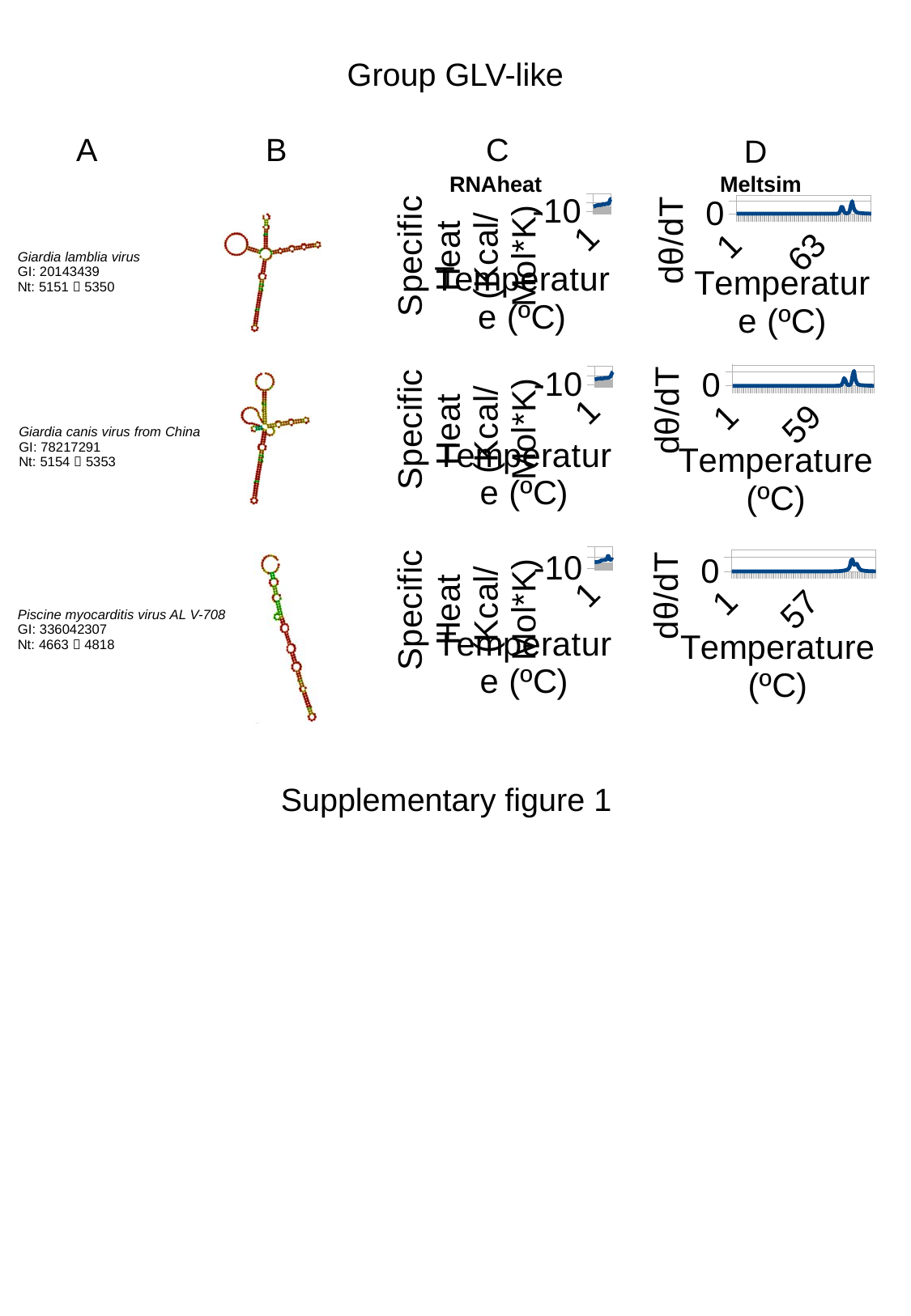

Group GLV-like
 B
A
 C
 D
RNAheat
Meltsim
### Chart
| Category | GLV |
|---|---|
| 1 | 1.24419 |
| 2 | 1.32524 |
| 3 | 1.41644 |
| 4 | 1.52079 |
| 5 | 1.63649 |
| 6 | 1.75881 |
| 7 | 1.88975 |
| 8 | 2.02648 |
| 9 | 2.17295 |
| 10 | 2.32337 |
| 11 | 2.47776 |
| 12 | 2.64012 |
| 13 | 2.81053 |
| 14 | 2.98406 |
| 15 | 3.15573 |
| 16 | 3.32249 |
| 17 | 3.48327 |
| 18 | 3.62991 |
| 19 | 3.76224 |
| 20 | 3.8791 |
| 21 | 3.98235 |
| 22 | 4.06467 |
| 23 | 4.12584 |
| 24 | 4.16719 |
| 25 | 4.18852 |
| 26 | 4.19273 |
| 27 | 4.18854 |
| 28 | 4.19521 |
| 29 | 4.24805 |
| 30 | 4.37492 |
| 31 | 4.56649 |
| 32 | 4.77833 |
| 33 | 4.95422 |
| 34 | 5.04306 |
| 35 | 5.00436 |
| 36 | 4.81807 |
| 37 | 4.57169 |
| 38 | 4.41287 |
| 39 | 4.38803 |
| 40 | 4.42499 |
| 41 | 4.47248 |
| 42 | 4.53004 |
| 43 | 4.59557 |
| 44 | 4.67356 |
| 45 | 4.76798 |
| 46 | 4.88232 |
| 47 | 5.02178 |
| 48 | 5.18937 |
| 49 | 5.38592 |
| 50 | 5.60999 |
| 51 | 5.85563 |
| 52 | 6.11512 |
| 53 | 6.37043 |
| 54 | 6.60501 |
| 55 | 6.79699 |
| 56 | 6.93107 |
| 57 | 6.99208 |
| 58 | 6.98051 |
| 59 | 6.90117 |
| 60 | 6.76997 |
| 61 | 6.60763 |
| 62 | 6.44013 |
| 63 | 6.28845 |
| 64 | 6.17444 |
| 65 | 6.11225 |
| 66 | 6.11151 |
| 67 | 6.17749 |
| 68 | 6.3096 |
| 69 | 6.498 |
| 70 | 6.72737 |
| 71 | 6.97891 |
| 72 | 7.22821 |
| 73 | 7.44829 |
| 74 | 7.61655 |
| 75 | 7.71513 |
| 76 | 7.73797 |
| 77 | 7.68545 |
| 78 | 7.56641 |
| 79 | 7.39635 |
| 80 | 7.20079 |
| 81 | 7.006 |
| 82 | 6.83856 |
| 83 | 6.72177 |
| 84 | 6.67688 |
| 85 | 6.72607 |
| 86 | 6.88746 |
| 87 | 7.1908 |
| 88 | 7.69599 |
| 89 | 8.41375 |
| 90 | 9.27897 |
| 91 | 10.2156 |
| 92 | 11.211 |
| 93 | 12.2697 |
| 94 | 13.3738 |
| 95 | 14.4831 |
| 96 | 15.5516 |
| 97 | 16.5316 |
| 98 | 17.3468 |
| 99 | 17.8892 |
| 100 | 18.0421 |
| 101 | 17.7323 |
### Chart
| Category | GLV |
|---|---|
| 1 | 8.0779e-06 |
| 2 | 8.543e-06 |
| 3 | 9.033e-06 |
| 4 | 9.5495e-06 |
| 5 | 1.0094e-05 |
| 6 | 1.0668e-05 |
| 7 | 1.1272e-05 |
| 8 | 1.191e-05 |
| 9 | 1.2583e-05 |
| 10 | 1.3293e-05 |
| 11 | 1.4043e-05 |
| 12 | 1.4834e-05 |
| 13 | 1.567e-05 |
| 14 | 1.6553e-05 |
| 15 | 1.7487e-05 |
| 16 | 1.8475e-05 |
| 17 | 1.9521e-05 |
| 18 | 2.0629e-05 |
| 19 | 2.1804e-05 |
| 20 | 2.305e-05 |
| 21 | 2.4373e-05 |
| 22 | 2.578e-05 |
| 23 | 2.7276e-05 |
| 24 | 2.887e-05 |
| 25 | 3.0569e-05 |
| 26 | 3.2383e-05 |
| 27 | 3.4321e-05 |
| 28 | 3.6396e-05 |
| 29 | 3.862e-05 |
| 30 | 4.1007e-05 |
| 31 | 4.3574e-05 |
| 32 | 4.6337e-05 |
| 33 | 4.9317e-05 |
| 34 | 5.2537e-05 |
| 35 | 5.6023e-05 |
| 36 | 5.9802e-05 |
| 37 | 6.3907e-05 |
| 38 | 6.8376e-05 |
| 39 | 7.3249e-05 |
| 40 | 7.8572e-05 |
| 41 | 8.4399e-05 |
| 42 | 9.0789e-05 |
| 43 | 9.781e-05 |
| 44 | 0.00010554 |
| 45 | 0.00011406 |
| 46 | 0.00012346 |
| 47 | 0.00013387 |
| 48 | 0.00014539 |
| 49 | 0.00015817 |
| 50 | 0.00017236 |
| 51 | 0.00018814 |
| 52 | 0.00020568 |
| 53 | 0.00022521 |
| 54 | 0.00024696 |
| 55 | 0.00027118 |
| 56 | 0.00029815 |
| 57 | 0.00032818 |
| 58 | 0.0003616 |
| 59 | 0.00039874 |
| 60 | 0.00043999 |
| 61 | 0.00048573 |
| 62 | 0.00053634 |
| 63 | 0.00059223 |
| 64 | 0.0006538 |
| 65 | 0.00072144 |
| 66 | 0.00079554 |
| 67 | 0.00087649 |
| 68 | 0.00096468 |
| 69 | 0.0010606 |
| 70 | 0.0011648 |
| 71 | 0.0012786 |
| 72 | 0.0014048 |
| 73 | 0.0015557 |
| 74 | 0.0017993 |
| 75 | 0.00259 |
| 76 | 0.0070719 |
| 77 | 0.033728 |
| 78 | 0.11273 |
| 79 | 0.10458 |
| 80 | 0.031219 |
| 81 | 0.010524 |
| 82 | 0.0083969 |
| 83 | 0.011632 |
| 84 | 0.032406 |
| 85 | 0.13391 |
| 86 | 0.20221 |
| 87 | 0.098885 |
| 88 | 0.049697 |
| 89 | 0.028632 |
| 90 | 0.018859 |
| 91 | 0.014363 |
| 92 | 0.011857 |
| 93 | 0.0099586 |
| 94 | 0.0082394 |
| 95 | 0.0066626 |
| 96 | 0.0052913 |
| 97 | 0.0041658 |
| 98 | 0.0032812 |
| 99 | 0.0026037 |
| 100 | 0.0020905 |
Giardia lamblia virus
GI: 20143439
Nt: 5151  5350
### Chart
| Category | GCV |
|---|---|
| 1 | 9.9942e-06 |
| 2 | 1.0687e-05 |
| 3 | 1.143e-05 |
| 4 | 1.2228e-05 |
| 5 | 1.3086e-05 |
| 6 | 1.4007e-05 |
| 7 | 1.4999e-05 |
| 8 | 1.6067e-05 |
| 9 | 1.7217e-05 |
| 10 | 1.8457e-05 |
| 11 | 1.9795e-05 |
| 12 | 2.124e-05 |
| 13 | 2.28e-05 |
| 14 | 2.4488e-05 |
| 15 | 2.6314e-05 |
| 16 | 2.8291e-05 |
| 17 | 3.0434e-05 |
| 18 | 3.2758e-05 |
| 19 | 3.5281e-05 |
| 20 | 3.8022e-05 |
| 21 | 4.1002e-05 |
| 22 | 4.4245e-05 |
| 23 | 4.7775e-05 |
| 24 | 5.1622e-05 |
| 25 | 5.5817e-05 |
| 26 | 6.0395e-05 |
| 27 | 6.5394e-05 |
| 28 | 7.0855e-05 |
| 29 | 7.6825e-05 |
| 30 | 8.3356e-05 |
| 31 | 9.0502e-05 |
| 32 | 9.8325e-05 |
| 33 | 0.00010689 |
| 34 | 0.00011627 |
| 35 | 0.00012655 |
| 36 | 0.00013781 |
| 37 | 0.00015014 |
| 38 | 0.00016364 |
| 39 | 0.00017842 |
| 40 | 0.00019458 |
| 41 | 0.00021225 |
| 42 | 0.00023155 |
| 43 | 0.0002526 |
| 44 | 0.00027553 |
| 45 | 0.00030048 |
| 46 | 0.00032756 |
| 47 | 0.00035691 |
| 48 | 0.00038864 |
| 49 | 0.00042285 |
| 50 | 0.00045965 |
| 51 | 0.00049911 |
| 52 | 0.00054129 |
| 53 | 0.00058624 |
| 54 | 0.00063398 |
| 55 | 0.00068452 |
| 56 | 0.00073788 |
| 57 | 0.00079405 |
| 58 | 0.00085307 |
| 59 | 0.00091501 |
| 60 | 0.00098001 |
| 61 | 0.0010483 |
| 62 | 0.0011203 |
| 63 | 0.0011966 |
| 64 | 0.0012781 |
| 65 | 0.0013658 |
| 66 | 0.0014614 |
| 67 | 0.0015666 |
| 68 | 0.0016838 |
| 69 | 0.0018158 |
| 70 | 0.0019658 |
| 71 | 0.0021376 |
| 72 | 0.002336 |
| 73 | 0.0025692 |
| 74 | 0.0028624 |
| 75 | 0.0033448 |
| 76 | 0.0048006 |
| 77 | 0.011974 |
| 78 | 0.046177 |
| 79 | 0.11226 |
| 80 | 0.082295 |
| 81 | 0.02569 |
| 82 | 0.010967 |
| 83 | 0.012439 |
| 84 | 0.036988 |
| 85 | 0.15676 |
| 86 | 0.21573 |
| 87 | 0.090687 |
| 88 | 0.037642 |
| 89 | 0.020252 |
| 90 | 0.014052 |
| 91 | 0.011344 |
| 92 | 0.0096063 |
| 93 | 0.0081085 |
| 94 | 0.0067183 |
| 95 | 0.0054695 |
| 96 | 0.0044052 |
| 97 | 0.0035369 |
| 98 | 0.0028485 |
| 99 | 0.0023108 |
| 100 | 0.0018927 |
### Chart
| Category | GCV |
|---|---|
| 1 | 1.9625 |
| 2 | 2.02036 |
| 3 | 2.0786 |
| 4 | 2.15068 |
| 5 | 2.2271 |
| 6 | 2.3108 |
| 7 | 2.40089 |
| 8 | 2.49645 |
| 9 | 2.59558 |
| 10 | 2.7042 |
| 11 | 2.82143 |
| 12 | 2.94635 |
| 13 | 3.08004 |
| 14 | 3.21959 |
| 15 | 3.35703 |
| 16 | 3.49133 |
| 17 | 3.61841 |
| 18 | 3.72605 |
| 19 | 3.812 |
| 20 | 3.88215 |
| 21 | 3.93327 |
| 22 | 3.96054 |
| 23 | 3.96011 |
| 24 | 3.93634 |
| 25 | 3.89108 |
| 26 | 3.83086 |
| 27 | 3.76285 |
| 28 | 3.70791 |
| 29 | 3.70241 |
| 30 | 3.77108 |
| 31 | 3.89828 |
| 32 | 4.03949 |
| 33 | 4.14749 |
| 34 | 4.17817 |
| 35 | 4.09648 |
| 36 | 3.88573 |
| 37 | 3.63148 |
| 38 | 3.46961 |
| 39 | 3.44336 |
| 40 | 3.48164 |
| 41 | 3.53539 |
| 42 | 3.59653 |
| 43 | 3.66898 |
| 44 | 3.75285 |
| 45 | 3.84989 |
| 46 | 3.95746 |
| 47 | 4.0762 |
| 48 | 4.20565 |
| 49 | 4.34592 |
| 50 | 4.49035 |
| 51 | 4.63728 |
| 52 | 4.78164 |
| 53 | 4.9183 |
| 54 | 5.04235 |
| 55 | 5.14652 |
| 56 | 5.22634 |
| 57 | 5.27929 |
| 58 | 5.30569 |
| 59 | 5.30646 |
| 60 | 5.28368 |
| 61 | 5.2441 |
| 62 | 5.194 |
| 63 | 5.14116 |
| 64 | 5.09085 |
| 65 | 5.05072 |
| 66 | 5.0259 |
| 67 | 5.02184 |
| 68 | 5.03872 |
| 69 | 5.07525 |
| 70 | 5.12831 |
| 71 | 5.19746 |
| 72 | 5.28102 |
| 73 | 5.37581 |
| 74 | 5.47831 |
| 75 | 5.58676 |
| 76 | 5.70258 |
| 77 | 5.82323 |
| 78 | 5.94218 |
| 79 | 6.05437 |
| 80 | 6.16081 |
| 81 | 6.26607 |
| 82 | 6.3783 |
| 83 | 6.50779 |
| 84 | 6.66865 |
| 85 | 6.87897 |
| 86 | 7.1575 |
| 87 | 7.53519 |
| 88 | 8.07408 |
| 89 | 8.7851 |
| 90 | 9.60208 |
| 91 | 10.4443 |
| 92 | 11.297 |
| 93 | 12.1666 |
| 94 | 13.0424 |
| 95 | 13.8991 |
| 96 | 14.7278 |
| 97 | 15.545 |
| 98 | 16.3551 |
| 99 | 17.1207 |
| 100 | 17.7549 |
| 101 | 18.136 |
Giardia canis virus from China
GI: 78217291
Nt: 5154  5353
### Chart
| Category | PISCINE |
|---|---|
| 1 | 1.18465 |
| 2 | 1.20906 |
| 3 | 1.23555 |
| 4 | 1.26363 |
| 5 | 1.29431 |
| 6 | 1.32711 |
| 7 | 1.36158 |
| 8 | 1.39626 |
| 9 | 1.42968 |
| 10 | 1.46231 |
| 11 | 1.49416 |
| 12 | 1.52669 |
| 13 | 1.55842 |
| 14 | 1.58884 |
| 15 | 1.61692 |
| 16 | 1.64667 |
| 17 | 1.67709 |
| 18 | 1.70921 |
| 19 | 1.74454 |
| 20 | 1.78569 |
| 21 | 1.83321 |
| 22 | 1.88871 |
| 23 | 1.9538 |
| 24 | 2.03014 |
| 25 | 2.11887 |
| 26 | 2.21959 |
| 27 | 2.33502 |
| 28 | 2.46374 |
| 29 | 2.60744 |
| 30 | 2.76416 |
| 31 | 2.93613 |
| 32 | 3.14471 |
| 33 | 3.39984 |
| 34 | 3.67792 |
| 35 | 3.93793 |
| 36 | 4.16409 |
| 37 | 4.35281 |
| 38 | 4.50373 |
| 39 | 4.6189 |
| 40 | 4.70261 |
| 41 | 4.75836 |
| 42 | 4.79245 |
| 43 | 4.81293 |
| 44 | 4.82848 |
| 45 | 4.84845 |
| 46 | 4.87872 |
| 47 | 4.91826 |
| 48 | 4.96382 |
| 49 | 5.01489 |
| 50 | 5.07292 |
| 51 | 5.13797 |
| 52 | 5.20842 |
| 53 | 5.28091 |
| 54 | 5.35204 |
| 55 | 5.41694 |
| 56 | 5.47241 |
| 57 | 5.51592 |
| 58 | 5.54693 |
| 59 | 5.5669 |
| 60 | 5.58024 |
| 61 | 5.5953 |
| 62 | 5.62302 |
| 63 | 5.67915 |
| 64 | 5.78315 |
| 65 | 5.95895 |
| 66 | 6.23358 |
| 67 | 6.6394 |
| 68 | 7.2044 |
| 69 | 7.94722 |
| 70 | 8.86648 |
| 71 | 9.92434 |
| 72 | 11.0294 |
| 73 | 12.0361 |
| 74 | 12.77 |
| 75 | 13.0848 |
| 76 | 12.9187 |
| 77 | 12.313 |
| 78 | 11.3906 |
| 79 | 10.3132 |
| 80 | 9.23235 |
| 81 | 8.25417 |
| 82 | 7.43314 |
| 83 | 6.78397 |
| 84 | 6.29884 |
| 85 | 5.9656 |
| 86 | 5.76504 |
| 87 | 5.674 |
| 88 | 5.67653 |
| 89 | 5.74958 |
| 90 | 5.85386 |
| 91 | 5.95563 |
| 92 | 6.04965 |
| 93 | 6.13423 |
| 94 | 6.19499 |
| 95 | 6.21153 |
| 96 | 6.17224 |
| 97 | 6.075 |
| 98 | 5.91892 |
| 99 | 5.70271 |
| 100 | 5.42768 |
| 101 | 5.10223 |
### Chart
| Category | PISCINE |
|---|---|
| 1 | 2.2522e-05 |
| 2 | 2.3859e-05 |
| 3 | 2.5273e-05 |
| 4 | 2.6768e-05 |
| 5 | 2.8348e-05 |
| 6 | 3.0018e-05 |
| 7 | 3.1784e-05 |
| 8 | 3.3651e-05 |
| 9 | 3.5623e-05 |
| 10 | 3.7708e-05 |
| 11 | 3.9911e-05 |
| 12 | 4.2238e-05 |
| 13 | 4.4697e-05 |
| 14 | 4.7294e-05 |
| 15 | 5.0035e-05 |
| 16 | 5.293e-05 |
| 17 | 5.5984e-05 |
| 18 | 5.9207e-05 |
| 19 | 6.2605e-05 |
| 20 | 6.6188e-05 |
| 21 | 6.9963e-05 |
| 22 | 7.3938e-05 |
| 23 | 7.8124e-05 |
| 24 | 8.2527e-05 |
| 25 | 8.7156e-05 |
| 26 | 9.202e-05 |
| 27 | 9.7127e-05 |
| 28 | 0.00010249 |
| 29 | 0.0001081 |
| 30 | 0.00011399 |
| 31 | 0.00012014 |
| 32 | 0.00012658 |
| 33 | 0.00013331 |
| 34 | 0.00014032 |
| 35 | 0.00014764 |
| 36 | 0.00015526 |
| 37 | 0.00016319 |
| 38 | 0.00017143 |
| 39 | 0.00017998 |
| 40 | 0.00018886 |
| 41 | 0.00019807 |
| 42 | 0.00020761 |
| 43 | 0.00021749 |
| 44 | 0.00022773 |
| 45 | 0.00023833 |
| 46 | 0.0002493 |
| 47 | 0.00026069 |
| 48 | 0.00027251 |
| 49 | 0.0002848 |
| 50 | 0.00029763 |
| 51 | 0.00031105 |
| 52 | 0.00032515 |
| 53 | 0.00034006 |
| 54 | 0.00035593 |
| 55 | 0.00037294 |
| 56 | 0.00039134 |
| 57 | 0.00041146 |
| 58 | 0.00043371 |
| 59 | 0.00045864 |
| 60 | 0.00048693 |
| 61 | 0.00051952 |
| 62 | 0.00055762 |
| 63 | 0.00060281 |
| 64 | 0.00065725 |
| 65 | 0.0007238 |
| 66 | 0.00080637 |
| 67 | 0.00091034 |
| 68 | 0.0010432 |
| 69 | 0.0012155 |
| 70 | 0.0014423 |
| 71 | 0.0017458 |
| 72 | 0.0021584 |
| 73 | 0.0027283 |
| 74 | 0.0035275 |
| 75 | 0.0046618 |
| 76 | 0.0062807 |
| 77 | 0.0085777 |
| 78 | 0.011759 |
| 79 | 0.015965 |
| 80 | 0.021384 |
| 81 | 0.030447 |
| 82 | 0.061389 |
| 83 | 0.15323 |
| 84 | 0.16678 |
| 85 | 0.091105 |
| 86 | 0.099272 |
| 87 | 0.10592 |
| 88 | 0.062922 |
| 89 | 0.031163 |
| 90 | 0.018887 |
| 91 | 0.013542 |
| 92 | 0.010177 |
| 93 | 0.0076716 |
| 94 | 0.0057918 |
| 95 | 0.004417 |
| 96 | 0.0034284 |
| 97 | 0.0027193 |
| 98 | 0.0022055 |
| 99 | 0.0018265 |
| 100 | 0.0015406 |
Piscine myocarditis virus AL V-708
GI: 336042307
Nt: 4663  4818
Supplementary figure 1
