# Supplementary material for: In silico single strand melting curve: a new approach to identify nucleic acid polymorphisms in Totiviridae
Source: BMC Bioinformatics. 2014 Jul 16;15(1):243. doi: 10.1186/1471-2105-15-243 (PMC4119202; doi:10.1186/1471-2105-15-243)
Supplement: Supplementary file 2 — Additional file 2: Figure S2: Regions with conserved RNA secondary structures identified in GaRV-like group and their respective melting curves. (A) Regions with secondary structures identified using RNAz software, from the alignment of ORF2 RNA sequences of GaRV-like group members. (B) Secondary structure calculated using RNAfold, corresponding to each conserved region identified by RNAz. (C) Melting curves calculated from the conserved region, using the software RNAheat which considers ssRNA denaturation. (D) Melting curves calculated from the conserved region, using the software MELTSIM which considers dsDNA denaturation. (PPT 302 KB) [file 12859_2013_6519_MOESM2_ESM.ppt]

## Slide 1
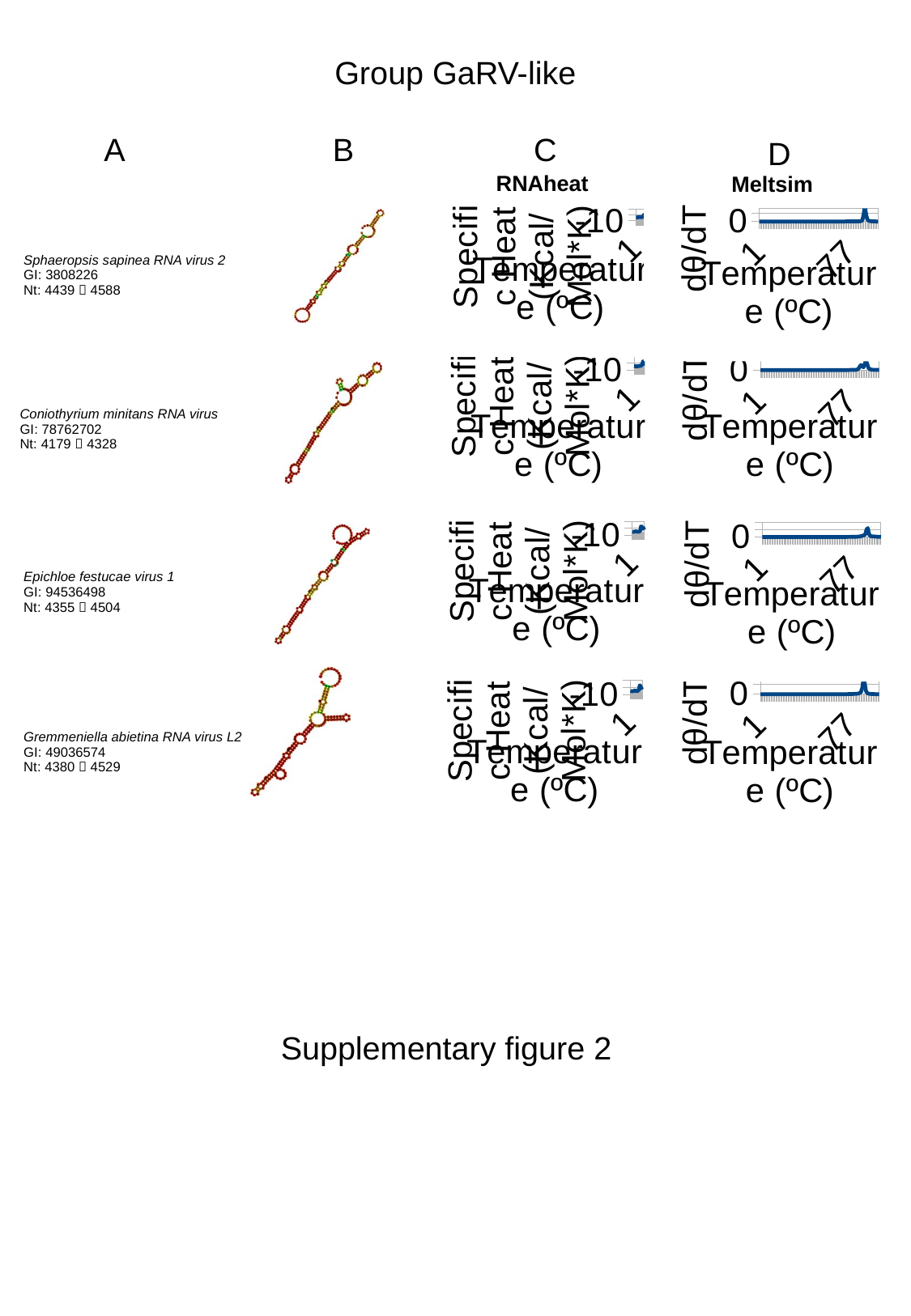

Group GaRV-like
A
 B
 C
 D
RNAheat
Meltsim
### Chart
| Category | SPHAERO |
|---|---|
| 1 | 1.50141 |
| 2 | 1.50452 |
| 3 | 1.51048 |
| 4 | 1.52031 |
| 5 | 1.53451 |
| 6 | 1.55412 |
| 7 | 1.58015 |
| 8 | 1.61317 |
| 9 | 1.65521 |
| 10 | 1.70489 |
| 11 | 1.77117 |
| 12 | 1.85918 |
| 13 | 1.96767 |
| 14 | 2.08535 |
| 15 | 2.20731 |
| 16 | 2.32856 |
| 17 | 2.44354 |
| 18 | 2.54509 |
| 19 | 2.63256 |
| 20 | 2.71038 |
| 21 | 2.77282 |
| 22 | 2.81819 |
| 23 | 2.84166 |
| 24 | 2.84768 |
| 25 | 2.83864 |
| 26 | 2.82479 |
| 27 | 2.8134 |
| 28 | 2.80815 |
| 29 | 2.8154 |
| 30 | 2.83318 |
| 31 | 2.8534 |
| 32 | 2.86201 |
| 33 | 2.84934 |
| 34 | 2.80556 |
| 35 | 2.7234 |
| 36 | 2.60679 |
| 37 | 2.49368 |
| 38 | 2.42305 |
| 39 | 2.4002 |
| 40 | 2.39676 |
| 41 | 2.40061 |
| 42 | 2.40937 |
| 43 | 2.42364 |
| 44 | 2.44373 |
| 45 | 2.4711 |
| 46 | 2.50829 |
| 47 | 2.55625 |
| 48 | 2.61451 |
| 49 | 2.68568 |
| 50 | 2.77325 |
| 51 | 2.87682 |
| 52 | 2.9999 |
| 53 | 3.1468 |
| 54 | 3.31971 |
| 55 | 3.52258 |
| 56 | 3.7611 |
| 57 | 4.04004 |
| 58 | 4.36581 |
| 59 | 4.74544 |
| 60 | 5.18478 |
| 61 | 5.68738 |
| 62 | 6.2538 |
| 63 | 6.87714 |
| 64 | 7.54057 |
| 65 | 8.2168 |
| 66 | 8.87361 |
| 67 | 9.47295 |
| 68 | 9.9683 |
| 69 | 10.3139 |
| 70 | 10.4854 |
| 71 | 10.4894 |
| 72 | 10.3563 |
| 73 | 10.131 |
| 74 | 9.86352 |
| 75 | 9.60143 |
| 76 | 9.38267 |
| 77 | 9.22338 |
| 78 | 9.12275 |
| 79 | 9.07429 |
| 80 | 9.07096 |
| 81 | 9.10222 |
| 82 | 9.15691 |
| 83 | 9.22372 |
| 84 | 9.29025 |
| 85 | 9.34493 |
| 86 | 9.37043 |
| 87 | 9.3454 |
| 88 | 9.25407 |
| 89 | 9.07371 |
| 90 | 8.77356 |
| 91 | 8.33893 |
| 92 | 7.79867 |
| 93 | 7.20166 |
| 94 | 6.56988 |
| 95 | 5.90253 |
| 96 | 5.21259 |
| 97 | 4.53853 |
| 98 | 3.9107 |
| 99 | 3.34582 |
| 100 | 2.85033 |
| 101 | 2.42546 |
### Chart
| Category | SPHAERO |
|---|---|
| 1 | 7.6241e-06 |
| 2 | 8.1187e-06 |
| 3 | 8.6452e-06 |
| 4 | 9.2058e-06 |
| 5 | 9.8031e-06 |
| 6 | 1.044e-05 |
| 7 | 1.1118e-05 |
| 8 | 1.1841e-05 |
| 9 | 1.2613e-05 |
| 10 | 1.3436e-05 |
| 11 | 1.4315e-05 |
| 12 | 1.5254e-05 |
| 13 | 1.6257e-05 |
| 14 | 1.7328e-05 |
| 15 | 1.8474e-05 |
| 16 | 1.97e-05 |
| 17 | 2.1012e-05 |
| 18 | 2.2417e-05 |
| 19 | 2.3922e-05 |
| 20 | 2.5535e-05 |
| 21 | 2.7264e-05 |
| 22 | 2.9119e-05 |
| 23 | 3.1109e-05 |
| 24 | 3.3246e-05 |
| 25 | 3.5541e-05 |
| 26 | 3.8008e-05 |
| 27 | 4.066e-05 |
| 28 | 4.3512e-05 |
| 29 | 4.6581e-05 |
| 30 | 4.9886e-05 |
| 31 | 5.3445e-05 |
| 32 | 5.728e-05 |
| 33 | 6.1414e-05 |
| 34 | 6.5872e-05 |
| 35 | 7.0684e-05 |
| 36 | 7.5878e-05 |
| 37 | 8.1488e-05 |
| 38 | 8.7551e-05 |
| 39 | 9.4107e-05 |
| 40 | 0.0001012 |
| 41 | 0.00010888 |
| 42 | 0.00011721 |
| 43 | 0.00012623 |
| 44 | 0.00013604 |
| 45 | 0.00014669 |
| 46 | 0.00015829 |
| 47 | 0.00017093 |
| 48 | 0.00018474 |
| 49 | 0.00019985 |
| 50 | 0.00021643 |
| 51 | 0.00023469 |
| 52 | 0.00025485 |
| 53 | 0.00027721 |
| 54 | 0.00030213 |
| 55 | 0.00033005 |
| 56 | 0.00036152 |
| 57 | 0.00039725 |
| 58 | 0.00043811 |
| 59 | 0.00048524 |
| 60 | 0.00054004 |
| 61 | 0.00060435 |
| 62 | 0.00068045 |
| 63 | 0.00077124 |
| 64 | 0.00088033 |
| 65 | 0.0010121 |
| 66 | 0.0011719 |
| 67 | 0.0013657 |
| 68 | 0.0016001 |
| 69 | 0.0018817 |
| 70 | 0.0022159 |
| 71 | 0.0026054 |
| 72 | 0.0030484 |
| 73 | 0.0035357 |
| 74 | 0.0040493 |
| 75 | 0.0045628 |
| 76 | 0.0050437 |
| 77 | 0.0054601 |
| 78 | 0.005789 |
| 79 | 0.0060239 |
| 80 | 0.0061795 |
| 81 | 0.006293 |
| 82 | 0.0064246 |
| 83 | 0.0066699 |
| 84 | 0.0072207 |
| 85 | 0.008653 |
| 86 | 0.013554 |
| 87 | 0.037708 |
| 88 | 0.17175 |
| 89 | 0.35389 |
| 90 | 0.16096 |
| 91 | 0.053017 |
| 92 | 0.023202 |
| 93 | 0.013192 |
| 94 | 0.0090353 |
| 95 | 0.0068761 |
| 96 | 0.0055202 |
| 97 | 0.0045535 |
| 98 | 0.0038123 |
| 99 | 0.0032218 |
| 100 | 0.0027415 |
Sphaeropsis sapinea RNA virus 2
GI: 3808226
Nt: 4439  4588
### Chart
| Category | CONIO |
|---|---|
| 1 | 1.0638e-05 |
| 2 | 1.1239e-05 |
| 3 | 1.1872e-05 |
| 4 | 1.2536e-05 |
| 5 | 1.3235e-05 |
| 6 | 1.397e-05 |
| 7 | 1.4743e-05 |
| 8 | 1.5555e-05 |
| 9 | 1.6409e-05 |
| 10 | 1.7307e-05 |
| 11 | 1.8252e-05 |
| 12 | 1.9245e-05 |
| 13 | 2.029e-05 |
| 14 | 2.139e-05 |
| 15 | 2.2547e-05 |
| 16 | 2.3765e-05 |
| 17 | 2.5048e-05 |
| 18 | 2.6399e-05 |
| 19 | 2.7822e-05 |
| 20 | 2.9321e-05 |
| 21 | 3.0902e-05 |
| 22 | 3.2569e-05 |
| 23 | 3.4328e-05 |
| 24 | 3.6184e-05 |
| 25 | 3.8145e-05 |
| 26 | 4.0215e-05 |
| 27 | 4.2404e-05 |
| 28 | 4.4719e-05 |
| 29 | 4.7168e-05 |
| 30 | 4.976e-05 |
| 31 | 5.2507e-05 |
| 32 | 5.5418e-05 |
| 33 | 5.8505e-05 |
| 34 | 6.1782e-05 |
| 35 | 6.5262e-05 |
| 36 | 6.8961e-05 |
| 37 | 7.2894e-05 |
| 38 | 7.7082e-05 |
| 39 | 8.1542e-05 |
| 40 | 8.6298e-05 |
| 41 | 9.1374e-05 |
| 42 | 9.6795e-05 |
| 43 | 0.00010259 |
| 44 | 0.0001088 |
| 45 | 0.00011545 |
| 46 | 0.00012259 |
| 47 | 0.00013026 |
| 48 | 0.00013852 |
| 49 | 0.00014742 |
| 50 | 0.00015704 |
| 51 | 0.00016745 |
| 52 | 0.00017874 |
| 53 | 0.00019104 |
| 54 | 0.00020445 |
| 55 | 0.00021914 |
| 56 | 0.00023529 |
| 57 | 0.00025311 |
| 58 | 0.00027287 |
| 59 | 0.0002949 |
| 60 | 0.00031957 |
| 61 | 0.0003474 |
| 62 | 0.00037896 |
| 63 | 0.000415 |
| 64 | 0.00045643 |
| 65 | 0.00050439 |
| 66 | 0.00056027 |
| 67 | 0.00062578 |
| 68 | 0.000703 |
| 69 | 0.00079444 |
| 70 | 0.0009031 |
| 71 | 0.0010325 |
| 72 | 0.0011865 |
| 73 | 0.0013696 |
| 74 | 0.0015864 |
| 75 | 0.001842 |
| 76 | 0.0021418 |
| 77 | 0.0024936 |
| 78 | 0.002915 |
| 79 | 0.0034622 |
| 80 | 0.0043464 |
| 81 | 0.0064018 |
| 82 | 0.01284 |
| 83 | 0.033736 |
| 84 | 0.07953 |
| 85 | 0.10371 |
| 86 | 0.071487 |
| 87 | 0.06062 |
| 88 | 0.12104 |
| 89 | 0.20328 |
| 90 | 0.12864 |
| 91 | 0.049746 |
| 92 | 0.022508 |
| 93 | 0.012679 |
| 94 | 0.0082044 |
| 95 | 0.0057965 |
| 96 | 0.0043447 |
| 97 | 0.0033957 |
| 98 | 0.0027374 |
| 99 | 0.0022597 |
| 100 | 0.0019009 |
### Chart
| Category | CONIO |
|---|---|
| 1 | 2.61223 |
| 2 | 2.57398 |
| 3 | 2.52291 |
| 4 | 2.45936 |
| 5 | 2.38707 |
| 6 | 2.30741 |
| 7 | 2.22759 |
| 8 | 2.14861 |
| 9 | 2.06901 |
| 10 | 1.99223 |
| 11 | 1.92128 |
| 12 | 1.85819 |
| 13 | 1.80108 |
| 14 | 1.74951 |
| 15 | 1.70255 |
| 16 | 1.66225 |
| 17 | 1.62667 |
| 18 | 1.59587 |
| 19 | 1.56989 |
| 20 | 1.5493 |
| 21 | 1.53364 |
| 22 | 1.52451 |
| 23 | 1.52043 |
| 24 | 1.52145 |
| 25 | 1.52917 |
| 26 | 1.55148 |
| 27 | 1.5932 |
| 28 | 1.65662 |
| 29 | 1.73672 |
| 30 | 1.83469 |
| 31 | 1.94121 |
| 32 | 2.04998 |
| 33 | 2.14665 |
| 34 | 2.21828 |
| 35 | 2.24642 |
| 36 | 2.22687 |
| 37 | 2.19312 |
| 38 | 2.19805 |
| 39 | 2.24854 |
| 40 | 2.31565 |
| 41 | 2.38177 |
| 42 | 2.448 |
| 43 | 2.51267 |
| 44 | 2.57853 |
| 45 | 2.64504 |
| 46 | 2.71246 |
| 47 | 2.78054 |
| 48 | 2.84676 |
| 49 | 2.91165 |
| 50 | 2.97606 |
| 51 | 3.04052 |
| 52 | 3.10561 |
| 53 | 3.17388 |
| 54 | 3.24648 |
| 55 | 3.32547 |
| 56 | 3.41432 |
| 57 | 3.51541 |
| 58 | 3.63232 |
| 59 | 3.76979 |
| 60 | 3.93351 |
| 61 | 4.12923 |
| 62 | 4.36393 |
| 63 | 4.64861 |
| 64 | 4.995 |
| 65 | 5.41525 |
| 66 | 5.92694 |
| 67 | 6.54921 |
| 68 | 7.29634 |
| 69 | 8.17299 |
| 70 | 9.17905 |
| 71 | 10.301 |
| 72 | 11.5015 |
| 73 | 12.7113 |
| 74 | 13.8336 |
| 75 | 14.7626 |
| 76 | 15.4061 |
| 77 | 15.7013 |
| 78 | 15.634 |
| 79 | 15.251 |
| 80 | 14.6426 |
| 81 | 13.9095 |
| 82 | 13.1392 |
| 83 | 12.3954 |
| 84 | 11.7175 |
| 85 | 11.1288 |
| 86 | 10.6362 |
| 87 | 10.2458 |
| 88 | 9.97892 |
| 89 | 9.80855 |
| 90 | 9.64744 |
| 91 | 9.41485 |
| 92 | 9.10456 |
| 93 | 8.72911 |
| 94 | 8.27796 |
| 95 | 7.73253 |
| 96 | 7.10429 |
| 97 | 6.43531 |
| 98 | 5.76271 |
| 99 | 5.10885 |
| 100 | 4.48713 |
| 101 | 3.91048 |
Coniothyrium minitans RNA virus
GI: 78762702
Nt: 4179  4328
### Chart
| Category | EPICHLOE |
|---|---|
| 1 | 0.694498 |
| 2 | 0.679829 |
| 3 | 0.665035 |
| 4 | 0.650116 |
| 5 | 0.637971 |
| 6 | 0.628631 |
| 7 | 0.619695 |
| 8 | 0.61361 |
| 9 | 0.609426 |
| 10 | 0.607166 |
| 11 | 0.611293 |
| 12 | 0.620389 |
| 13 | 0.634009 |
| 14 | 0.654696 |
| 15 | 0.682021 |
| 16 | 0.716054 |
| 17 | 0.76039 |
| 18 | 0.813618 |
| 19 | 0.876842 |
| 20 | 0.948632 |
| 21 | 1.03265 |
| 22 | 1.12389 |
| 23 | 1.22293 |
| 24 | 1.3252 |
| 25 | 1.42761 |
| 26 | 1.5286 |
| 27 | 1.62241 |
| 28 | 1.7053 |
| 29 | 1.774 |
| 30 | 1.82574 |
| 31 | 1.8561 |
| 32 | 1.86487 |
| 33 | 1.85078 |
| 34 | 1.812 |
| 35 | 1.75096 |
| 36 | 1.6723 |
| 37 | 1.5823 |
| 38 | 1.4879 |
| 39 | 1.39612 |
| 40 | 1.30861 |
| 41 | 1.22543 |
| 42 | 1.14936 |
| 43 | 1.08102 |
| 44 | 1.02325 |
| 45 | 0.973672 |
| 46 | 0.934299 |
| 47 | 0.905791 |
| 48 | 0.886855 |
| 49 | 0.877864 |
| 50 | 0.878632 |
| 51 | 0.889812 |
| 52 | 0.910089 |
| 53 | 0.941815 |
| 54 | 0.985093 |
| 55 | 1.04174 |
| 56 | 1.11245 |
| 57 | 1.1985 |
| 58 | 1.30462 |
| 59 | 1.43533 |
| 60 | 1.59344 |
| 61 | 1.78764 |
| 62 | 2.02667 |
| 63 | 2.32452 |
| 64 | 2.69783 |
| 65 | 3.17063 |
| 66 | 3.77252 |
| 67 | 4.54293 |
| 68 | 5.52394 |
| 69 | 6.75203 |
| 70 | 8.24125 |
| 71 | 9.96137 |
| 72 | 11.8069 |
| 73 | 13.5881 |
| 74 | 15.0588 |
| 75 | 15.9984 |
| 76 | 16.3017 |
| 77 | 16.0213 |
| 78 | 15.3368 |
| 79 | 14.474 |
| 80 | 13.6226 |
| 81 | 12.8968 |
| 82 | 12.3406 |
| 83 | 11.9525 |
| 84 | 11.7077 |
| 85 | 11.5788 |
| 86 | 11.5375 |
| 87 | 11.5694 |
| 88 | 11.6835 |
| 89 | 11.8426 |
| 90 | 11.9545 |
| 91 | 11.941 |
| 92 | 11.8039 |
| 93 | 11.563 |
| 94 | 11.2098 |
| 95 | 10.7194 |
| 96 | 10.0918 |
| 97 | 9.36069 |
| 98 | 8.56429 |
| 99 | 7.73625 |
| 100 | 6.90668 |
| 101 | 6.10563 |
### Chart
| Category | EPICHLOE |
|---|---|
| 1 | 8.041e-06 |
| 2 | 8.4948e-06 |
| 3 | 8.9715e-06 |
| 4 | 9.4721e-06 |
| 5 | 9.9978e-06 |
| 6 | 1.055e-05 |
| 7 | 1.113e-05 |
| 8 | 1.1739e-05 |
| 9 | 1.2378e-05 |
| 10 | 1.3049e-05 |
| 11 | 1.3755e-05 |
| 12 | 1.4496e-05 |
| 13 | 1.5274e-05 |
| 14 | 1.6093e-05 |
| 15 | 1.6953e-05 |
| 16 | 1.7857e-05 |
| 17 | 1.8809e-05 |
| 18 | 1.981e-05 |
| 19 | 2.0864e-05 |
| 20 | 2.1974e-05 |
| 21 | 2.3144e-05 |
| 22 | 2.4378e-05 |
| 23 | 2.568e-05 |
| 24 | 2.7054e-05 |
| 25 | 2.8507e-05 |
| 26 | 3.0044e-05 |
| 27 | 3.167e-05 |
| 28 | 3.3394e-05 |
| 29 | 3.5222e-05 |
| 30 | 3.7163e-05 |
| 31 | 3.9227e-05 |
| 32 | 4.1424e-05 |
| 33 | 4.3764e-05 |
| 34 | 4.6263e-05 |
| 35 | 4.8933e-05 |
| 36 | 5.1791e-05 |
| 37 | 5.4855e-05 |
| 38 | 5.8147e-05 |
| 39 | 6.1689e-05 |
| 40 | 6.5508e-05 |
| 41 | 6.9635e-05 |
| 42 | 7.4106e-05 |
| 43 | 7.8961e-05 |
| 44 | 8.4247e-05 |
| 45 | 9.0021e-05 |
| 46 | 9.635e-05 |
| 47 | 0.00010331 |
| 48 | 0.000111 |
| 49 | 0.00011953 |
| 50 | 0.00012905 |
| 51 | 0.00013972 |
| 52 | 0.00015176 |
| 53 | 0.00016544 |
| 54 | 0.00018108 |
| 55 | 0.00019912 |
| 56 | 0.00022008 |
| 57 | 0.00024464 |
| 58 | 0.00027366 |
| 59 | 0.00030825 |
| 60 | 0.00034979 |
| 61 | 0.00040008 |
| 62 | 0.00046136 |
| 63 | 0.00053645 |
| 64 | 0.00062884 |
| 65 | 0.0007428 |
| 66 | 0.0008834 |
| 67 | 0.0010565 |
| 68 | 0.0012684 |
| 69 | 0.0015257 |
| 70 | 0.0018341 |
| 71 | 0.0021971 |
| 72 | 0.0026151 |
| 73 | 0.003083 |
| 74 | 0.0035905 |
| 75 | 0.0041239 |
| 76 | 0.0046718 |
| 77 | 0.0052391 |
| 78 | 0.0058693 |
| 79 | 0.0066829 |
| 80 | 0.0079388 |
| 81 | 0.010122 |
| 82 | 0.013999 |
| 83 | 0.020386 |
| 84 | 0.029202 |
| 85 | 0.038645 |
| 86 | 0.04928 |
| 87 | 0.082032 |
| 88 | 0.20884 |
| 89 | 0.24269 |
| 90 | 0.083511 |
| 91 | 0.030111 |
| 92 | 0.020019 |
| 93 | 0.017024 |
| 94 | 0.014726 |
| 95 | 0.012263 |
| 96 | 0.0098232 |
| 97 | 0.0076699 |
| 98 | 0.0059224 |
| 99 | 0.0045759 |
| 100 | 0.0035663 |
Epichloe festucae virus 1
GI: 94536498
Nt: 4355  4504
### Chart
| Category | GREMME |
|---|---|
| 1 | 3.9207e-06 |
| 2 | 4.161e-06 |
| 3 | 4.415e-06 |
| 4 | 4.6835e-06 |
| 5 | 4.9672e-06 |
| 6 | 5.267e-06 |
| 7 | 5.5839e-06 |
| 8 | 5.9189e-06 |
| 9 | 6.2731e-06 |
| 10 | 6.6476e-06 |
| 11 | 7.0438e-06 |
| 12 | 7.4628e-06 |
| 13 | 7.9063e-06 |
| 14 | 8.3758e-06 |
| 15 | 8.8729e-06 |
| 16 | 9.3995e-06 |
| 17 | 9.9576e-06 |
| 18 | 1.0549e-05 |
| 19 | 1.1177e-05 |
| 20 | 1.1843e-05 |
| 21 | 1.2551e-05 |
| 22 | 1.3302e-05 |
| 23 | 1.4102e-05 |
| 24 | 1.4952e-05 |
| 25 | 1.5858e-05 |
| 26 | 1.6823e-05 |
| 27 | 1.7853e-05 |
| 28 | 1.8951e-05 |
| 29 | 2.0126e-05 |
| 30 | 2.1381e-05 |
| 31 | 2.2726e-05 |
| 32 | 2.4167e-05 |
| 33 | 2.5713e-05 |
| 34 | 2.7374e-05 |
| 35 | 2.916e-05 |
| 36 | 3.1084e-05 |
| 37 | 3.316e-05 |
| 38 | 3.5402e-05 |
| 39 | 3.7827e-05 |
| 40 | 4.0457e-05 |
| 41 | 4.3312e-05 |
| 42 | 4.6419e-05 |
| 43 | 4.9809e-05 |
| 44 | 5.3515e-05 |
| 45 | 5.758e-05 |
| 46 | 6.2051e-05 |
| 47 | 6.6987e-05 |
| 48 | 7.2457e-05 |
| 49 | 7.8544e-05 |
| 50 | 8.5354e-05 |
| 51 | 9.3011e-05 |
| 52 | 0.00010167 |
| 53 | 0.00011154 |
| 54 | 0.00012285 |
| 55 | 0.00013592 |
| 56 | 0.00015114 |
| 57 | 0.00016903 |
| 58 | 0.00019023 |
| 59 | 0.00021556 |
| 60 | 0.00024611 |
| 61 | 0.00028324 |
| 62 | 0.00032872 |
| 63 | 0.00038481 |
| 64 | 0.00045443 |
| 65 | 0.00054126 |
| 66 | 0.00064995 |
| 67 | 0.00078631 |
| 68 | 0.00095745 |
| 69 | 0.001172 |
| 70 | 0.0014398 |
| 71 | 0.001772 |
| 72 | 0.0021802 |
| 73 | 0.0026752 |
| 74 | 0.0032652 |
| 75 | 0.0039532 |
| 76 | 0.0047353 |
| 77 | 0.0055995 |
| 78 | 0.0065306 |
| 79 | 0.0075236 |
| 80 | 0.0086201 |
| 81 | 0.0099892 |
| 82 | 0.012125 |
| 83 | 0.016355 |
| 84 | 0.026192 |
| 85 | 0.051435 |
| 86 | 0.12974 |
| 87 | 0.30981 |
| 88 | 0.21908 |
| 89 | 0.065786 |
| 90 | 0.025515 |
| 91 | 0.013279 |
| 92 | 0.0081902 |
| 93 | 0.0056874 |
| 94 | 0.0042991 |
| 95 | 0.0034433 |
| 96 | 0.0028654 |
| 97 | 0.0024451 |
| 98 | 0.0021215 |
| 99 | 0.0018616 |
| 100 | 0.0016464 |
### Chart
| Category | GREMME |
|---|---|
| 1 | 1.32898 |
| 2 | 1.26595 |
| 3 | 1.20628 |
| 4 | 1.14854 |
| 5 | 1.09325 |
| 6 | 1.04093 |
| 7 | 0.992583 |
| 8 | 0.947285 |
| 9 | 0.90703 |
| 10 | 0.868926 |
| 11 | 0.836454 |
| 12 | 0.813146 |
| 13 | 0.797111 |
| 14 | 0.783439 |
| 15 | 0.771656 |
| 16 | 0.762785 |
| 17 | 0.756356 |
| 18 | 0.753912 |
| 19 | 0.752957 |
| 20 | 0.760128 |
| 21 | 0.776021 |
| 22 | 0.798161 |
| 23 | 0.823521 |
| 24 | 0.856265 |
| 25 | 0.89543 |
| 26 | 0.940558 |
| 27 | 0.994837 |
| 28 | 1.06045 |
| 29 | 1.13645 |
| 30 | 1.22453 |
| 31 | 1.3322 |
| 32 | 1.51534 |
| 33 | 1.80076 |
| 34 | 2.1318 |
| 35 | 2.40818 |
| 36 | 2.58852 |
| 37 | 2.67886 |
| 38 | 2.69348 |
| 39 | 2.64844 |
| 40 | 2.54863 |
| 41 | 2.40635 |
| 42 | 2.23927 |
| 43 | 2.06751 |
| 44 | 1.90895 |
| 45 | 1.77921 |
| 46 | 1.68607 |
| 47 | 1.62041 |
| 48 | 1.56574 |
| 49 | 1.51407 |
| 50 | 1.46514 |
| 51 | 1.42122 |
| 52 | 1.38435 |
| 53 | 1.35517 |
| 54 | 1.33573 |
| 55 | 1.32585 |
| 56 | 1.32819 |
| 57 | 1.34142 |
| 58 | 1.37025 |
| 59 | 1.41309 |
| 60 | 1.47268 |
| 61 | 1.5506 |
| 62 | 1.65053 |
| 63 | 1.77543 |
| 64 | 1.93021 |
| 65 | 2.12027 |
| 66 | 2.35197 |
| 67 | 2.63615 |
| 68 | 2.98619 |
| 69 | 3.41825 |
| 70 | 3.95214 |
| 71 | 4.61371 |
| 72 | 5.43211 |
| 73 | 6.43515 |
| 74 | 7.6419 |
| 75 | 9.05242 |
| 76 | 10.6289 |
| 77 | 12.2772 |
| 78 | 13.8474 |
| 79 | 15.1666 |
| 80 | 16.0894 |
| 81 | 16.5427 |
| 82 | 16.5457 |
| 83 | 16.1873 |
| 84 | 15.5826 |
| 85 | 14.8367 |
| 86 | 14.0192 |
| 87 | 13.173 |
| 88 | 12.3384 |
| 89 | 11.5305 |
| 90 | 10.736 |
| 91 | 9.95043 |
| 92 | 9.23373 |
| 93 | 8.67802 |
| 94 | 8.27172 |
| 95 | 7.90183 |
| 96 | 7.47811 |
| 97 | 7.01442 |
| 98 | 6.54372 |
| 99 | 6.09542 |
| 100 | 5.68968 |
| 101 | 5.33967 |Gremmeniella abietina RNA virus L2
GI: 49036574
Nt: 4380  4529
Supplementary figure 2
