# Supplementary figures and images for: In silico single strand melting curve: a new approach to identify nucleic acid polymorphisms in Totiviridae
Source: BMC Bioinformatics. 2014 Jul 16;15(1):243. doi: 10.1186/1471-2105-15-243 (PMC4119202; doi:10.1186/1471-2105-15-243)

## Slide 1
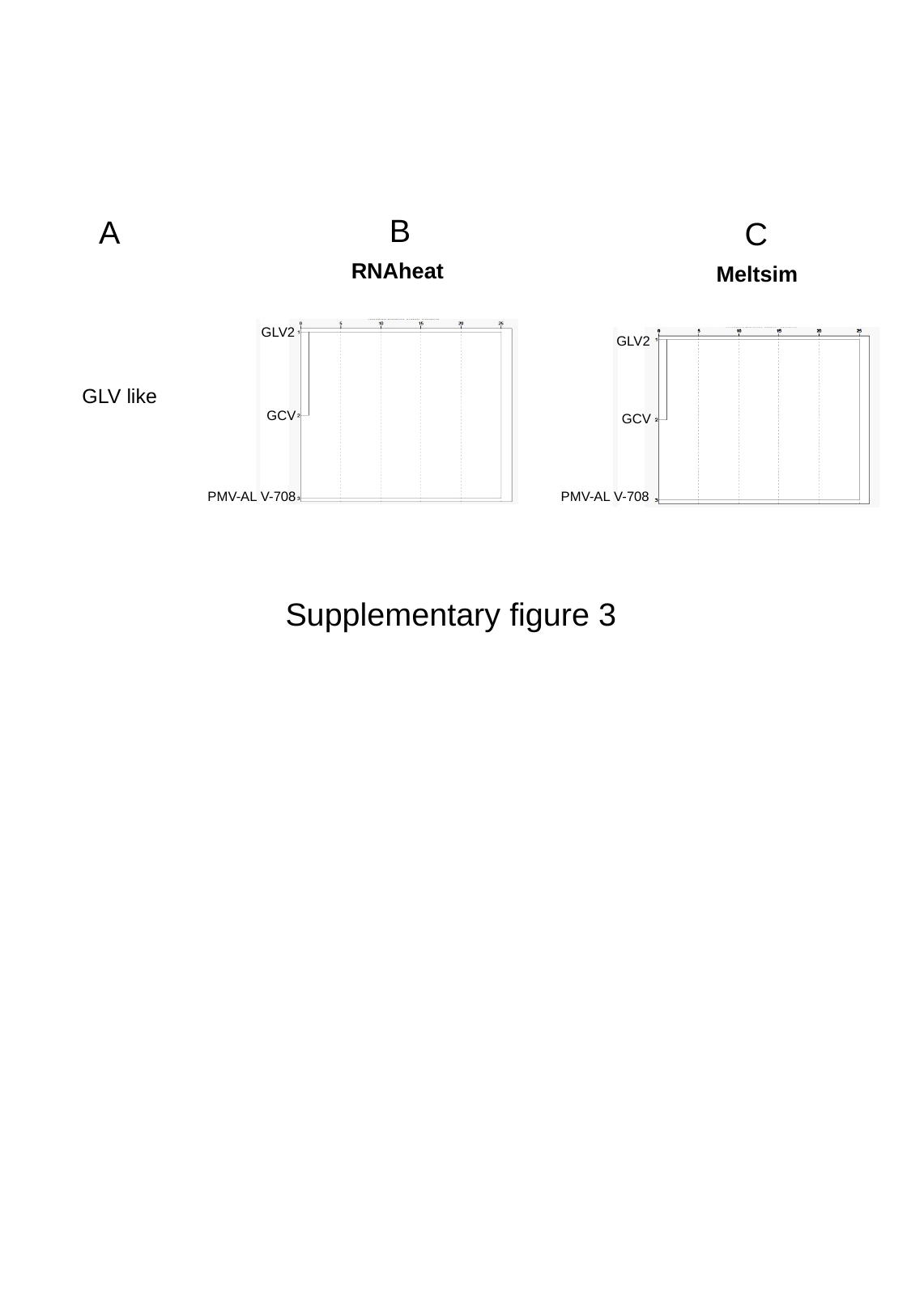

B
A
C
RNAheat
Meltsim
GLV2
GLV2
GLV like
GCV
GCV
PMV-AL V-708
PMV-AL V-708
Supplementary figure 3

Supplement: Supplementary file 3 — Additional file 3: Figure S3: Cluster analysis and dendogram of GLV-like group. The curves generated for each sequence were compared and clustered using a statistical inference. The proximity between individuals of groups indicated in the column (A) is due exclusively to the similarity between the melting curves generated in silico. Columns (B) and (C) shows the dendograms calculated from the curves generated by the programs RNAheat and MELSTSIM for the members of GLV group. (PPT 355 KB) [file 12859_2013_6519_MOESM3_ESM.ppt]
